# Multistep sequence-controlled supramolecular polymerization by the combination of multiple self-assembly motifs

**DOI:** 10.1016/j.isci.2023.106023

**Published:** 2023-01-23

**Authors:** Hui Li, Shenghui Rao, Ying Yang, Fenfen Xu, Zhe Huang, Xiaohui Huang, Zhu Zhu, Shengyong Liu, Zhelin Zhang, Wei Tian

**Affiliations:** 1 Jiangxi Provincial Key Laboratory of Functional Molecular Materials Chemistry, School of Chemistry and Chemical Engineering, Jiangxi University of Science and Technology, Ganzhou 341000, P. R. China; 2 Shaanxi Key Laboratory of Macromolecular Science and Technology, School of Science, Northwestern Polytechnical University, Xi’an 710072, P. R. China

**Keywords:** Polymer chemistry, Supramolecular chemistry, Molecular self-assembly

## Abstract

The precise sequence control of polymer chain is an important research topic of polymer chemistry. Although some methods such as iterative synthesis and supramolecular polymerization have been developed to fabricate sequence-controllable polymer, it is still a great challenge to consecutively prepare multiple supramolecular polymers with different sequence structures. In this work, through the reasonable utilization of assembly motifs, we integrated multiple host-guest recognitions and metal coordination interactions to prepare different sequence-controlled supramolecular polymers by a multistep assembly strategy. This research provides inspiration for the design and preparation of supramolecular polymers with different sequence structures.

## Highlights

• Multiple supramolecular polymers were prepared by a multistep assembly strategy
• Supramolecular polymers with different structures show different stimuli responsiveness
• Different supramolecular polymer materials show different fluorescence emission

## Graphical abstract

**Figure:**
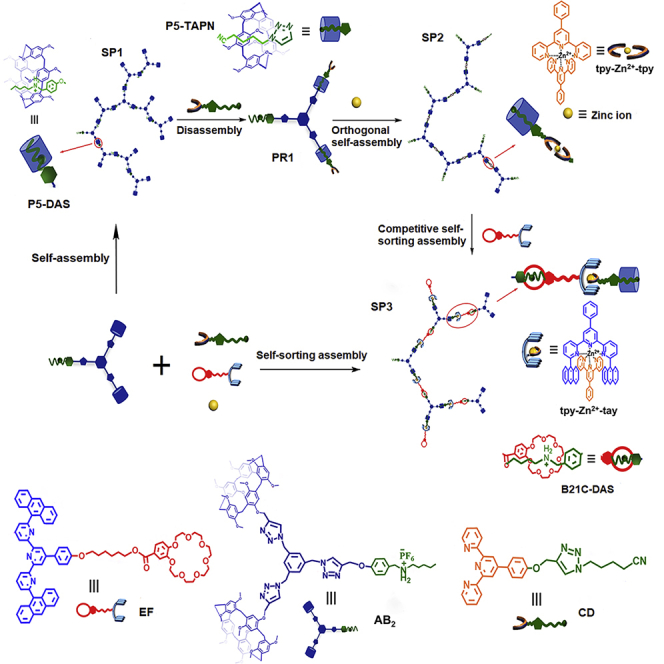

## Introduction

The precise sequence control of polymer chain is a great challenge in polymer synthesis, which gives rise to structural and functional diversity.^1^^,^^2^^,^^3^^,^^4^^,^^5^ The sequence-controlled polymers are generally prepared by using iterative chemistry technology or sequential monomer addition method,^6^^,^^7^^,^^8^^,^^9^^,^^10^^,^^11^^,^^12^^,^^13^ such as the stepwise iterative synthesis for peptide on a solid-polymer support. However, iterative synthesis generally needs very high reaction yield and undergo an annoying purification process to remove error structures. Supramolecular polymerization opens up another way to develop sequence-controlled polymers.^14^^,^^15^^,^^16^ Various supramolecular polymerization methods have been developed to fabricate well-defined supramolecular polymers.^17^^,^^18^^,^^19^^,^^20^^,^^21^ Among these polymerization methodologies, self-assembly with single noncovalent interaction has been widely utilized to prepare supramolecular polymers. Compared to the self-assembly with one type of noncovalent interaction, orthogonal self-assembly incorporating two or multiple noncovalent interactions can endow the resulting supramolecular polymers with structural diversity and more specific properties.^18^ In a process of orthogonal self-assembly, each noncovalent interaction does not affect each other. Compared to the noninterference features of noncovalent interactions in the process of orthogonal self-assembly, competitive self-sorting assembly describes the fact that the existence of competitive noncovalent interactions and the process of self-sorting assembly.^21^ By means of the competitive self-sorting assembly, supramolecular polymers can easily achieve the sequence reorganization and architecture transformation, such as the structural conversion from hyperbranched supramolecular homopolymer to the hyperbranched supramolecular copolymer.^21^

Although various supramolecular polymers with different sequences and topological structures have been constructed under the direction of these assembly methods,^22^^,^^23^^,^^24^^,^^25^^,^^26^^,^^27^^,^^28^^,^^29^^,^^30^^,^^31^^,^^32^^,^^33^^,^^34^^,^^35^^,^^36^^,^^37^^,^^38^ consecutively preparing multiple sequence-controlled supramolecular polymers is still a great challenge. Alternatively, combining the merits of different polymerization methods and using a multistep assembly strategy may effectively solve the problem. Multistep assembly strategy can avoid the tedious synthesis and purification to some degree, and also endows the resulting polymers with the architecture and function diversities. To construct supramolecular polymers with different sequence structures by the multistep assembly, multiple different supramolecular forces with high specificities are required. Crown ethers and pillararenes-based host-guest recognition pairs have been applied to construct supramolecular assemblies.^19^ On the other hand, terpyridine (tpy) and its complementary ligand pairs have been also reported to form metal coordination structures with metal ions.^27^ Herein, we combine different polymerization methods and apply multiple host-guest recognitions and metal coordination interactions to develop different sequence-controlled supramolecular polymers by a multistep assembly strategy. These different supramolecular forces with high specificities include crown ethers and pillararenes-based host-guest pairs, terpyridine and its complementary ligand pairs.

**Scheme 1:**
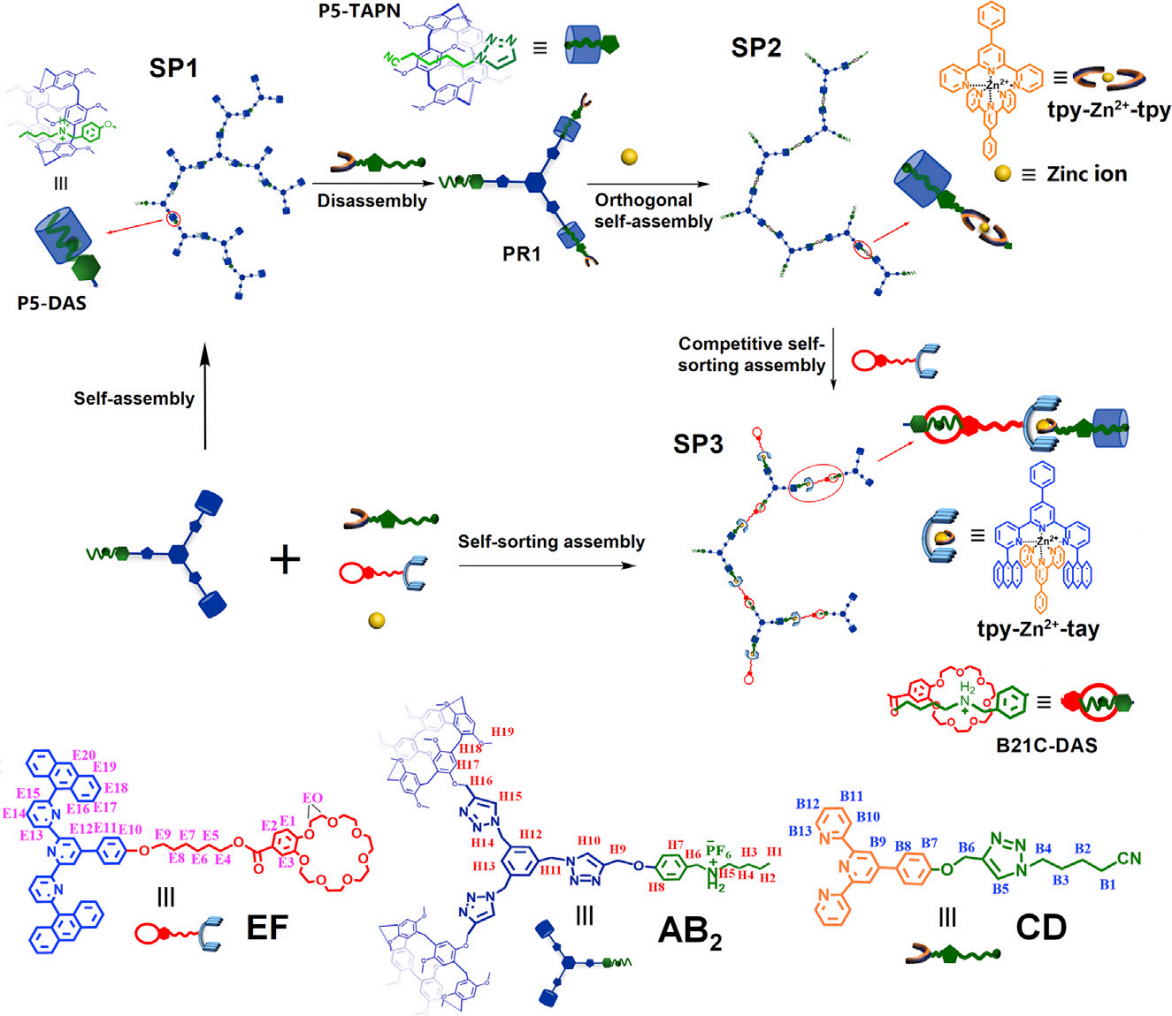
Schematic illustration of multistep self-assembly The construction of the supramolecular homopolymer SP1, the disassembly of SP1, the construction of supramolecular copolymer SP2 based on orthogonal self-assembly, and the architecture transformation from supramolecular copolymer SP2 to SP3 based on competitive self-sorting assembly.

We designed and synthesized three heterotopic monomers as follows (Scheme 1). The heterotritopic **AB**_**2**_, which contained two pillar[5]arene groups (**P5**) and an alkylammonium salt group (**DAS**); the heteroditopic **CD** consisted of a terpyridine (**tpy**) moiety and an alkyl chain guest (**TAPN**) moiety, and the heteroditopic **EF** beared a 6,6″-anthracyl-substituted tpy (**tay**) group and a crown ether (**B21C**) group. The AB_2_ could self-organize to form supramolecular hyperbranched homopolymer (**SP1**) by the host-guest interaction of P5-DAS. When adding the monomer CD to the solution of SP1, the SP1 could disassemble into pseudorotaxane **PR1** by competitive host-guest interaction because of the stronger binding of P5-TAPN. After Zn(OTf)_2_ was added to the solution of pseudorotaxane PR1, the PR1 transformed into a new supramolecular hyperbranched copolymer **SP2** based on the orthogonal noncovalent interaction of P5-TAPN and tpy-Zn^2+^-tpy. Moreover, as the tpy and tay formed complementary ligands pair that could undergo spontaneous heteroleptic complexation with zinc ion, when monomer EF was further added into the solution of SP2, the SP2 could further transform into a new supramolecular hyperbranched copolymer **SP3** with new sequence structure through the P5-TAPN, B21C-DAS, and tpy-Zn^2+^-tay binding events based on the competitive self-sorting assembly.

## Results and discussion

### NMR spectra and UV-Vis study

**Scheme 2:**
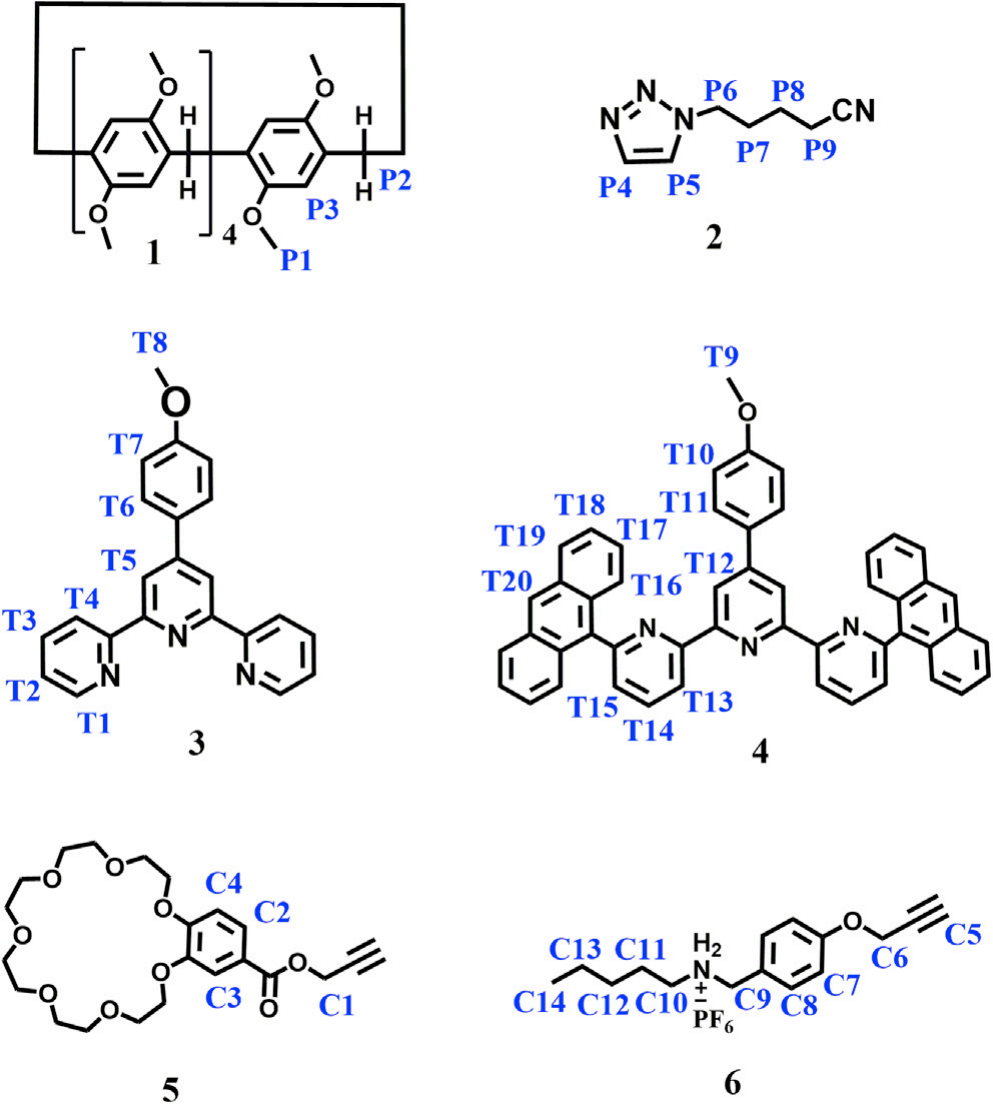
The structures of the model molecules 1–6

To study the multistep assembly process, we synthesized six model compounds 1–6 (Scheme 2). Firstly, a series of ^1^H NMR spectra containing two model compounds were recorded (Figures S1–S6, ESI†). When equimolar 1 and 6 were mixed in CDCl_3_ solution, the signals of protons C_10_, C_11_, C_12,_ C_13_, C_14_ on 6 were shifted upfield (Figure S1, ESI†), suggesting that the alkylammonium salt of M6 entered into the cavity of pillararene in CDCl_3_ solution and formed the complex of P5-DAS.^39^ It’s worth noting that the proton peak shifts of 1 + 6 were not observed in CDCl_3_-CD_3_COCD_3_ (Figure S2, ESI†), indicating that the P5 could not bind the DAS or had extremely weak binding in the mixed solvent of CDCl_3_-CD_3_COCD_3_. Cation–π interaction was dependent on the type of solvent,^40^ the cation–π interaction should be severely weakened in the polar mixed solvent. The host–guest complexation of 1 and 2 was then studied. The ^1^H NMR spectrum showed that P5 could bind strongly TAPN in solution (Figure S3, ESI†).^28^ The host-guest recognition between 5 and 6 was also studied, the complex ^1^H NMR verified that the exchanging interaction between B21C and DAS was slow (Figure S4, ESI†).^41^ The metal coordination of tpy-Zn^2+^-tpy was clearly observed when compound 3 and Zn(OTf)_2_ were mixed in solution (Figure S5E and Figure S6, ESI†). In comparison with the metal coordination of tpy-Zn^2+^-tpy, the formation ratio for tay-Zn^2+^-tay was much slower, and an incomplete conversion (41%) was observed for 4 days (Figure S5A),^27^ presumably because of the increased bulkiness of the substituents at terpyridyl 6,6″-positions. However, when equimolar 3 + 4+Zn(OTf)_2_ was mixed in solution, the coordination protons of tpy-Zn^2+^-tpy rapidly disappeared and new chemical shifts of coordination protons were observed on the ^1^H NMR timescale, implying that the homoleptic coordination of tpy-Zn^2+^-tpy was disrupted and complementary tpy and tay ligands spontaneously self-assembled into heteroleptic tpy-Zn^2+^-tay structure with zinc ion (Figure S5C, ESI†).^27^ Furthermore, the heteroleptic complexation of tpy-Zn^2+^-tay was further verified by the ESI-MS peak at *m*/*z* = 547.1676 originated from [Zn34]^2+^ (Figure S7, ESI†), which provided direct evidence of the heteroleptic tpy-Zn^2+^-tay structure. These experimental results supported that the tpy-Zn^2+^-tay structure was more stable than that of tpy-Zn^2+^-tpy in CDCl_3_-CD_3_COCD_3_ (3:1, v/v).

The self-sorting binding among different model compounds were then investigated, a series of samples containing different noncovalent interactions were prepared and their ^1^H NMR spectra verified the self-sorting binding between P5-TAPN and tpy-Zn^2+^-tay (Figure S8, ESI†), between P5-TAPN and B21C-DAS (Figure S9, ESI†), between B21C-DAS and tpy-Zn^2+^-tay (Figure S10, ESI†). Finally, the ^1^H NMR spectrum of 1 + 2+3 + 4+5 + 6+Zn(OTf)_2_ revealed that the self-sorting binding indeed occurred in CDCl_3_-CD_3_COCD_3_ (Figure S11, ESI†). That is, P5 combined with TAPN, tpy bound tay in the presence of Zn^2+^, and B21C bound DAS in CDCl_3_–CD_3_COCD_3_ solution, respectively.

**Figure 1:**
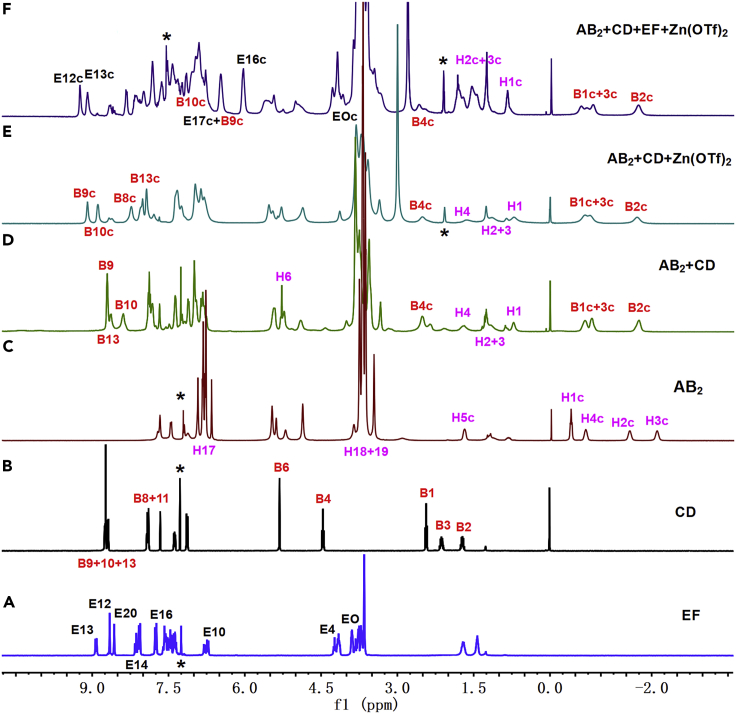
^1^H NMR spectra (400 MHz, CDCl_3_, 15 mM, 298 K) (A) EF, (B) CD, (C) AB_2_, (D) AB_2_+CD; ^1^H NMR spectra (400 MHz, CDCl_3_-CD_3_COCD_3_ = 3:1, v/v, 15 mM, 298 K) of (E) AB_2_+CD + Zn(OTf)_2_, and (F) AB_2_+CD + EF + Zn(OTf)_2_. Proton signals of complexed monomers are designated as c.

We then studied the multistep self-assembly of monomers. The self-assembly of AB_2_ in chloroform-*d* solution was first studied by ^1^H NMR. As shown in Figure 1C, the alkyl protons H_1-5_ of AB_2_ exhibited significant upfield shifts (−2.08, −1.54, −0.68, −0.39, 1.70 ppm) in CDCl_3_ solution, suggesting that the alkyl wheel of the DAS group on AB_2_ fully inserted into the hole of the P5 group on AB_2_. By means of the COSY NMR analysis (Figures S12–S14, ESI†), the ^1^H NMR of AB_2_ was identified. The ^1^H NMR of AB_2_ also revealed that the P5-DAS binding interaction was a fast-exchange interaction in solution. The ^1^H NMR spectra gradually broadened as the concentration of AB_2_ increased (Figure S15, ESI†), implying that the AB_2_ self-assembled into supramolecular homopolymer SP1 at high concentrations. Considering that the pillar[5]arene can encapsulate neutral guest molecule,^42^^,^^43^ adding monomer CD to the solution of AB_2_ may induce the disassembly of SP1. As showed in Figure 1D, when CD was added to the solution of AB_2_, the original complexed protons on AB_2_ disappeared (H1c-5c) and new complexed protons B1c-4c (2.55, −0.66, −0.79, −1.71 ppm) on CD were observed simultaneously on the NMR timescale, indicating that the supramolecular homopolymer SP1 was disassembled into [3]pseudorotaxane PR1. Considering terpyridyl can coordinate with metal ion, the PR1 may further transform into supramolecular hyperbranched copolymer SP2 by adding Zn(OTf)_2_. Owing to the poor solubility of Zn(OTf)_2_ in CDCl_3_, the Zn(OTf)_2_ was firstly dissolved in deuterated acetone and was then added into the solution of PR1. The protons B8-10 on CD shifted downfield and the proton B13 on CD shifted upfield, implying the occurrence of metal coordination between terpyridyl and zinc ion (Figure 1E).^44^^,^^45^ Meanwhile, the ^1^H NMR also revealed that the coordination of tpy-Zn^2+^-tpy did not interfere with the binding of P5-TAPN. The orthogonal noncovalent interactions between tpy-Zn^2+^-tpy and P5-TAPN and the broadened ^1^H NMR spectra at high concentrations (Figure S16, ESI†) supported that the PR1 transformed into a new supramolecular hyperbranched copolymer SP2. According to the previous report,^27^ in the presence of metal ion, tay can spontaneously form heteroleptic complex with tpy in organic solvent. Thus, the SP2 was expected to further transform into a new supramolecular copolymer SP3 when adding EF into the solution of SP2 through the competitive self-sorting assembly. As shown in Figure 1F, the original complexed protons (B9c, B10c) on CD disappeared, and new complexed protons (B9c, B10c, E16c) shifted upfield, verifying the dissociation of the tpy-Zn^2+^-tpy and the formation of new tpy-Zn^2+^-tay coordination structure.^27^ Meanwhile, the complexation of B21C-DAS was also observed (H1c, H2c, H3c, EOc), which manifested the occurrence of host-guest recognition between B21C and DAS.^46^^,^^47^ In addition, the ^1^H NMR also revealed that the metal coordination tpy-Zn^2+^-tay did not affect the host-guest interaction of P5-TAPN. Moreover, the ^1^H NMR obviously broadened at high concentration (Figure S17, ESI†). The above ^1^H NMR analysis and the concentration-dependent ^1^H NMR spectra supported that the SP2 transformed into another sequence-controlled SP3 when adding EF into the solution of SP2.

**Figure 2:**
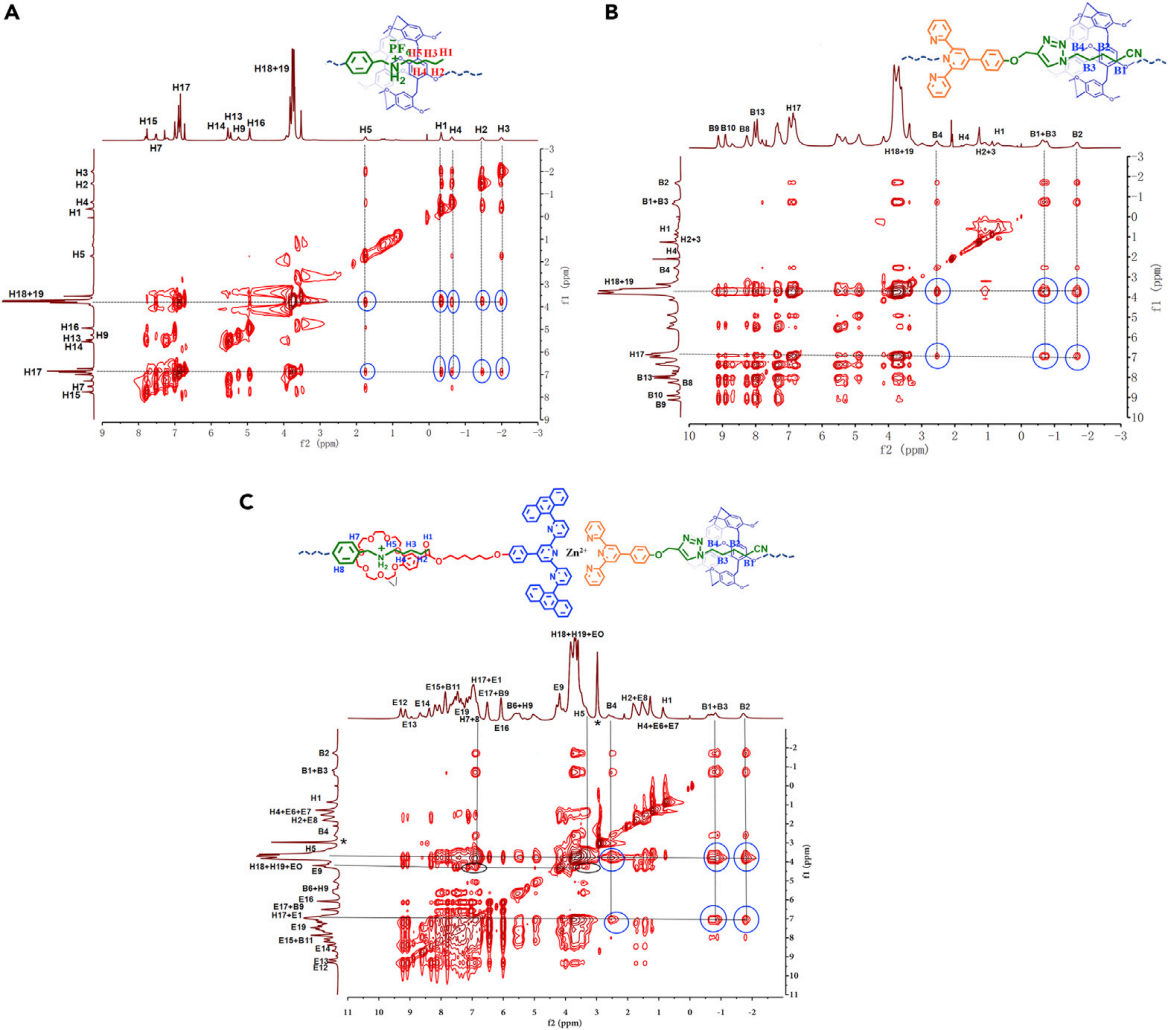
2D NOESY NMR  spectra (A) AB_2_ in CDCl_3_, (B) AB_2_+CD + Zn(OTf)_2_ in CDCl_3_-CD_3_COCD_3_ (3/1, v/v), (C) AB_2_+CD + EF + Zn(OTf)_2_ in CDCl_3_-CD_3_COCD_3_ (3/1, v/v, 400 MHz, 60 mM, 298K).

2D NOESY NMR was also conducted to investigate the multistep assembly of monomers. For the AB_2_ system, the strong correlations among H1-5 and H17-19 on AB_2_ verified that the DAS group of AB_2_ entered the P5 cavity of AB_2_ (Figure 2A, ESI†). After adding the monomer CD and Zn(OTf)_2_ into the solution of AB_2_, the correlations among H1-5 and H17-19 disappeared and new correlations among B1-4 on CD and H17-19 on AB_2_ were observed (Figure 2B, ESI†), indicating that the DAS moiety of AB_2_ was squeezed out the cavity of P5 by TAPN of CD due to the stronger binding between P5 and TAPN. After further adding EF to the solution of AB_2_+CD + Zn(OTf)_2_, the correlations among B1-4 and H17-19 were still observed, and new correlations among H5, H7, H8 of AB_2_ and EO of EF were also found in the 2D NOESY spectrum (Figure 2C, ESI†), verifying the coexist of host-guest interactions P5-TAPN and B21C-DAS in solution.

**Figure 3:**
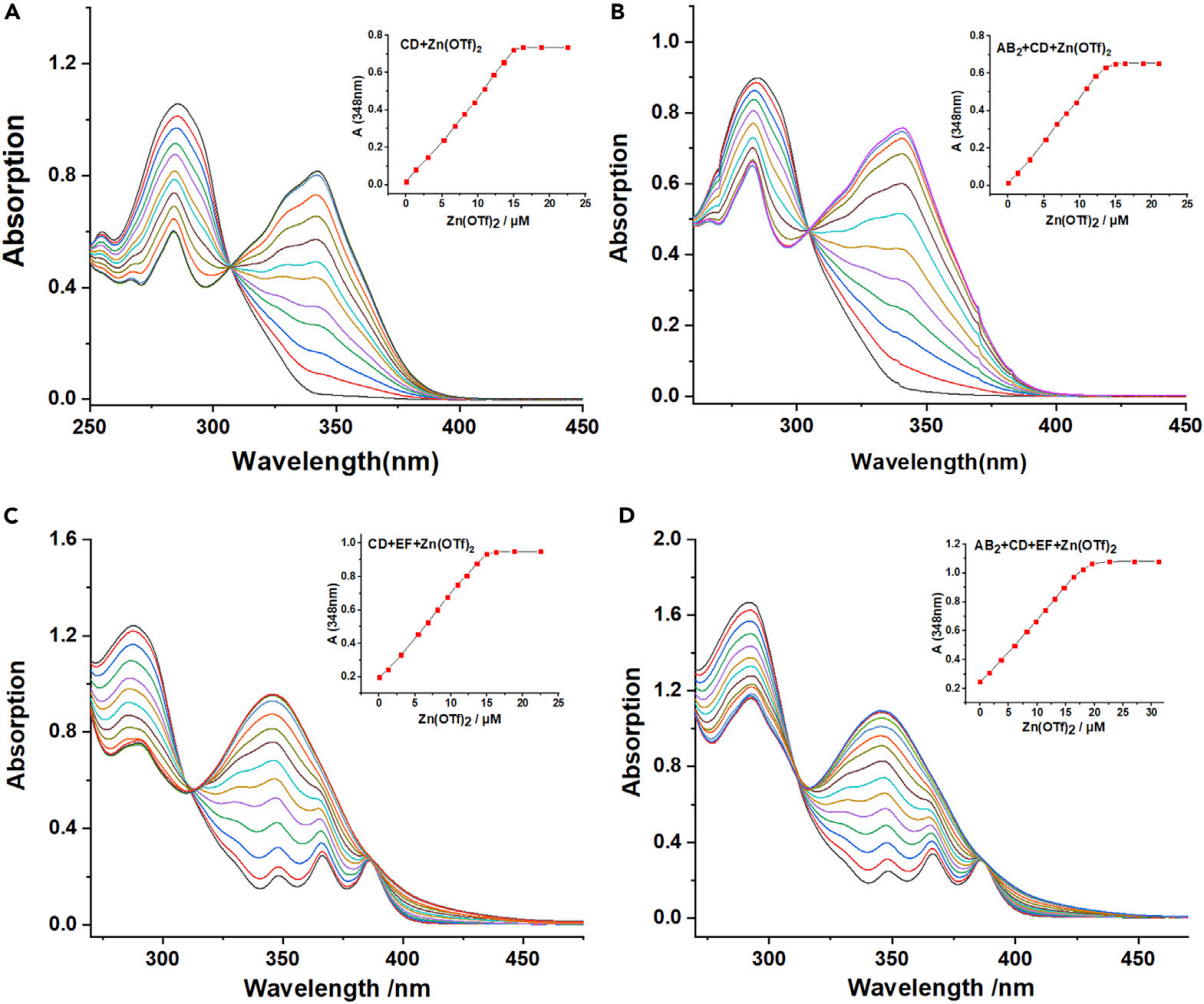
Changes in the UV-Vis absorption spectra Stepwise adding Zn(OTf)_2_ (2 mM) to the solutions of (A) 0.015 mM CD, (B) 0.015 mM AB_2_+CD, (C) 0.015 mM CD + EF, and (D) 0.020 mM AB_2_+CD + EF (chloroform versus acetone=3:1, v/v, 298K).

UV-Vis titrations were also performed to investigate the multistep assembly of monomers. The titrations were firstly performed through titrating Zn(OTf)_2_ into the solution of CD (Figure 3A), the UV-Vis titration curves exhibited an isosbestic point at 308 nm, implying that the free tpy gradually transformed into the coordination structure.^44^ The titration curve has a maximum absorbance at 343 nm when the molar ratio of Zn(OTf)_2_ versus CD was 1 : 2. The variation of the absorbance versus the concentration of Zn(OTf)_2_ was plotted (inset), which verified the formation of the tpy-Zn^2+^-tpy coordination structure in the solution. The titration curve of AB_2_+CD + Zn(OTf)_2_ was similar to that of CD + Zn(OTf)_2_ (Figure 3B), indicating that the host-guest interaction P5-TAPN did not interfere with the metal coordination of tpy-Zn^2+^-tpy. Similarly, the titrations of CD + EF and AB_2_+CD + EF were also performed by adding Zn(OTf)_2_. As shown in Figure 3C, the titration curves of CD + EF + Zn(OTf)_2_ revealed an isosbestic point at 313 nm and a maximum absorbance at 346 nm when the molar ratio of Zn(OTf)_2_: CD: EF is 1:1:1. The variation of the absorbance versus the concentration of Zn(OTf)_2_ was plotted (inset), which verified the generation of heteroleptic complex tpy-Zn^2+^-tay. As shown in Figure 3D, the titration plots of AB_2_+CD + EF + Zn(OTf)_2_ were similar to those of CD + EF + Zn(OTf)_2_, and a titration endpoint was achieved when the molar ratio was 1:2:2:2 (AB_2_:CD: EF: Zn(OTf)_2_), indicating that the B21C-DAS and P5-TAPN host-guest recognitions didn’t affect the coordination of tpy-Zn^2+^-tay. The binding constants of tpy-Zn^2+^-tpy and tpy-Zn^2+^-tay were then calculated by job plot method of UV-Vis (Figures S18–S21, ESI†), and the data verified that the binding constant of tpy-Zn^2+^-tay was higher than that of tpy-Zn^2+^-tpy, in accordance with the mass spectra and ^1^H NMR analysis (Figures S5–S7, ESI†). The binding constants of host-guest interactions P5-DAS, P5-TAPN, B21C-DAS were also measured by reference methods (Figures S22–S25, ESI†).

**Figure 4:**
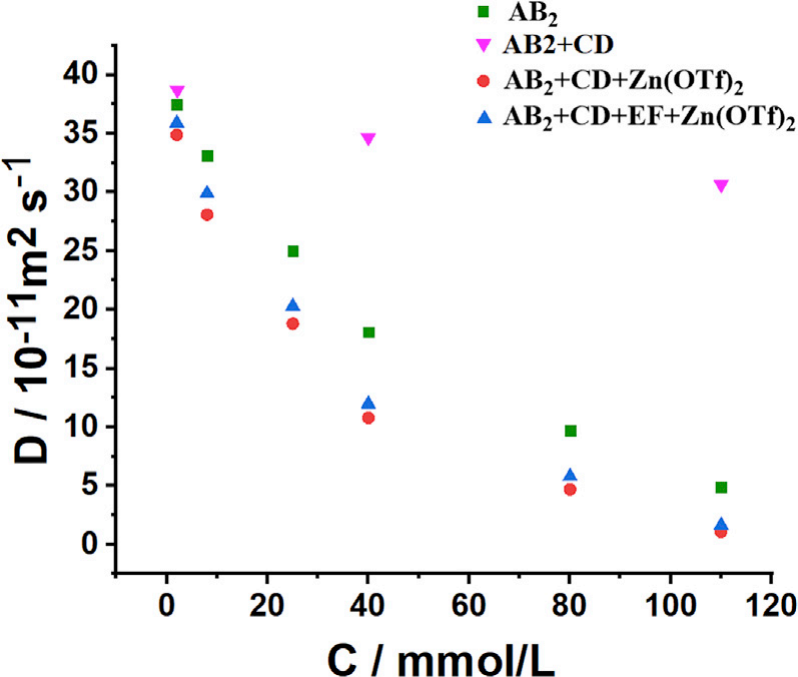
Average weight diffusion coefficients of AB_2_, AB_2_+CD, AB_2_+CD + Zn(OTf)_2_, and AB_2_+CD + EF + Zn(OTf)_2_ versus the concentrations of AB_2_ at 298K

DOSY NMR were then performed to further investigate the supramolecular polymerization of multistep assembly. For the AB_2_ system, when the concentration of AB_2_ gradually increased (2–110 mM), the measured weight average diffusion coefficient (*D*) ranged from 3.75 × 10^−10^ to 4.88 × 10^−11^ m^2^ s^−1^ (Figures 4 and S26), verified the concentration-dependent characteristic of supramolecular polymerization and the formation of supramolecular homopolymer SP1 at high concentration.^48^ When CD was added into the SP1 constructed by self-assembly of AB_2_ (AB_2_ = 110 mM), the *D* value increased dramatically from 4.88× 10^−11^ to 3.05× 10^−10^ m^2^ s^−1^ (Figures 4 and S27), implying that SP1 disassembled into low molecular weight PR1. When Zn(OTf)_2_ was subsequently added into the PR1, the *D* value reduced considerably from 3.05× 10^−10^ to 1.08× 10^−11^ m^2^ s^−1^, suggesting the formation of a new supramolecular copolymer SP2 (Figures 4 and S28). After EF was further added to the solution of SP2, the *D* value changed from 1.08× 10^−11^ to 1.65× 10^−11^ m^2^ s^−1^, supporting that the SP2 transformed into another supramolecular copolymer SP3. Both the *D* values gradually increased as the concentrations diluted (Figures 4 and S29), which further verified the reversibility of noncovalent interactions and concentration-dependence feature of supramolecular polymerization.^49^ It is worth noting that the *D* value for SP2 was slightly larger than that of SP3 at the same concentration of AB_2_ (110 mM). It can be reasonably explained as follows: The molecular weight and polymerization degree of supramolecular polymers depend on the binding constants of noncovalent bonding and monomers concentrations. The SP3 was constructed based on three types of noncovalent interactions, the minimum binding constant among P5-TAPN, tpy-Zn^2+^-tay, and B21C-DAS mainly determined the polymerization degree and molecular weight of SP3. Although the binding constant of tpy-Zn^2+^-tay on SP3 was larger than that of tpy-Zn^2+^-tpy on SP2, the binding constant of B21C-DAS on SP3 was smaller than those of P5-TAPN and tpy-Zn^2+^-tpy on SP2. Thus, it is reasonable that the molecular weight of SP3 was smaller than that of SP2, which corresponds to the experimental *D* values of SP2 and SP3.

### Study of viscosity, DLS, and SEM

**Figure 5:**
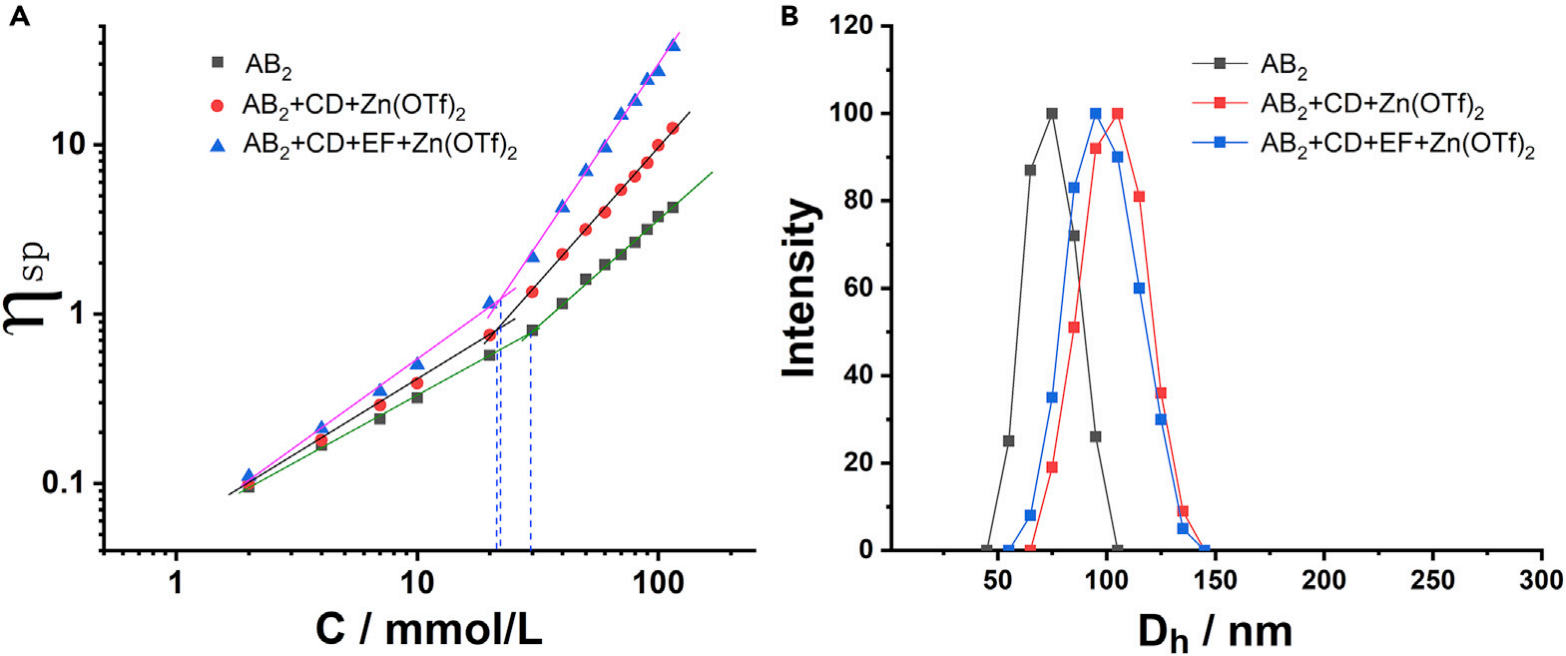
Specific viscosities and hydrodynamic diameter distributions (A) Specific viscosities of AB_2_, AB_2_+CD + Zn(OTf)_2_, and AB_2_+CD + EF + Zn(OTf)_2_ against the concentrations of AB_2_ at 298K; (B) the hydrodynamic diameter distributions of AB_2_, AB_2_+CD + Zn(OTf)_2_, and AB_2_+CD + EF + Zn(OTf)_2_ in solutions (AB_2_ = 120 mM, 298 K).

Viscosity measurements were also conducted to investigate the multistep supramolecular polymerization. The specific viscosity of the AB_2_, AB_2_+CD + Zn(OTf)_2_, and AB_2_+CD + EF + Zn(OTf)_2_ against the concentration of AB_2_ (bilogarithmic plot) was plotted in Figure 5A. At the same concentrations, both the AB_2_+CD + Zn(OTf)_2_ and AB_2_+CD + EF + Zn(OTf)_2_ had higher specific viscosities than that of AB_2_ system, which could ascribe to the higher molecular weight of SP2 and SP3. In addition, both the critical polymerization concentrations of SP2 and SP3 (the intersection of two lines) had lower values than that of AB_2_, this result may derive from the lower binding constant of P5-DAS on AB_2_. At low concentrations, supramolecular oligomers were main species,^50^^,^^51^ and supramolecular oligomers gradually transformed into supramolecular polymers when the concentrations were above the critical polymerization concentrations in the AB_2_, AB_2_+CD + Zn(OTf)_2_, and AB_2_+CD + EF + Zn(OTf)_2_ systems.

**Figure 6:**
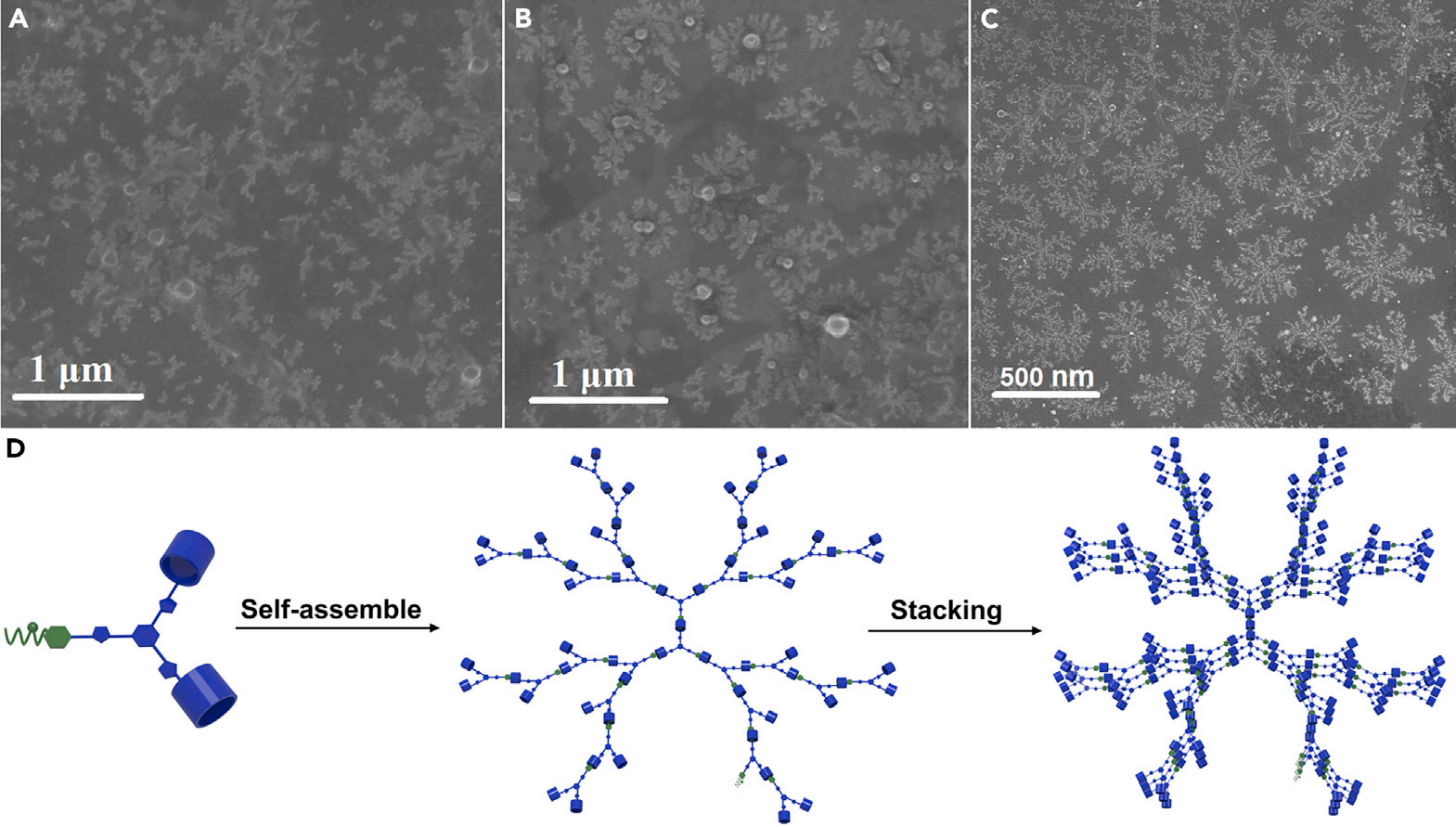
Representative SEM images of the supramolecular polymers (The samples were prepared from 35 mM solutions) (A–C)SP1, (b)SP2, (c)SP3. (D) The graphical illustration of the formation of macro-sized branched structures.

The sizes of supramolecular polymers in solutions were then investigated by dynamic light scattering (DLS) experiments (Figure 5B). The SP1 constructed by self-assembly of AB_2_ had a hydrodynamic diameter *(D*_*h*_) of 73 nm in solution (120 mM). A higher *D*_*h*_ value of 105 nm was observed for the SP2 solution of AB_2_+CD + Zn(OTf)_2_. The higher *D*_*h*_ of AB_2_+CD + Zn(OTf)_2_ was reasonable because the binding constants of P5-TAPN and tpy-Zn^2+^-tpy were both larger than that of P5-DAS, the higher binding constants generally resulted in the larger sizes of supramolecular aggregates at same concentration. When EF was added to the solution of AB_2_+CD + Zn(OTf)_2_, the *D*_*h*_ value became 96 nm, the relatively smaller *D*_*h*_ value for AB_2_+CD + EF + Zn(OTf)_2_ was speculated to derive from the smaller binding constant of B21C-DAS compared to those of P5-TAPN and tpy-Zn^2+^-tpy. The morphologies of SP1-SP3 were observed by SEM. As shown in Figures 6A, 6B, and 6C, widespread branched structures with ranging from tens of nanometers to hundreds of nanometers were observed in all representative SEM images of the SP1, SP2, and SP3 samples. To eliminate the possibility of samples crystallization, XRD experiments were performed. The XRD analysis indicated that the samples of SP1-SP3 are amorphous and eliminate the possibility of samples crystallization (Figure S30). Compared with the SEM image of SP1, the SEM images of SP2 and SP3 look more regular. The binding constant of noncovalent interactions may have an influence on the morphology of supramolecular polymers. The binding constant of P5-DAS on SP1 was smaller than those of SP2 and SP3, which may induce the smaller sizes of SP1 as observed in the SEM image. Moreover, a close inspection of the branched structures in SEM images showed that the larger branched structures contained the stacking of smaller branched structures. It was reasonable that smaller branches may superimpose on each other to form macro-sized branched aggregates when the samples changed from solutions to dry solids during the preparation of SEM samples, the cartoon representation of the formation of branched morphology was depicted in Figure 6D.

### Stimuli-responsiveness of supramolecular polymers

Finally, we investigated the stimuli-responsiveness of supramolecular polymers. The SP1 exhibited competitive guest responsiveness due to the stronger binding between TAPN and P5 (Figure 1D). Similarly, the SP2 also exhibited competitive guest responsiveness, when butanedinitrile was added into the solution of SP2, the SP2 disassembled into low molecular weight oligomers (Figure S31, ESI†) due to the stronger binding of P5-butanedinitrile. Besides the P5-TAPN binding could be manipulated, the metal coordination could also be adjusted, when compound 4 was added into the solution of SP2 (Figure S32, ESI†), the ^1^H NMR revealed the disassembly of SP2 as the tpy-Zn^2+^-tay binding was tighter than that of tpy-Zn^2+^-tpy. Compared to the SP2 system, the SP3 contained three types of noncovalent interactions, which may exhibit more stimuli-responsiveness. First, the butanedinitrile could also induce the disassembly of SP3 (Figure S33, ESI†). Second, it was found that the SP3 exhibited K^+^ responsiveness. When KPF_6_ was added to the SP3 solution, the ^1^H NMR obviously became sharper (Figure S34, ESI†), implying the disassembly of SP3.^47^ After another crown ether (B18C6) of smaller ring was added to the solution, the ^1^H NMR became complicated again as the B18C6 could capture K^+^ and the host-guest binding of B21C-DAS was recovered, which induced the reformation of SP3.

**Figure 7:**
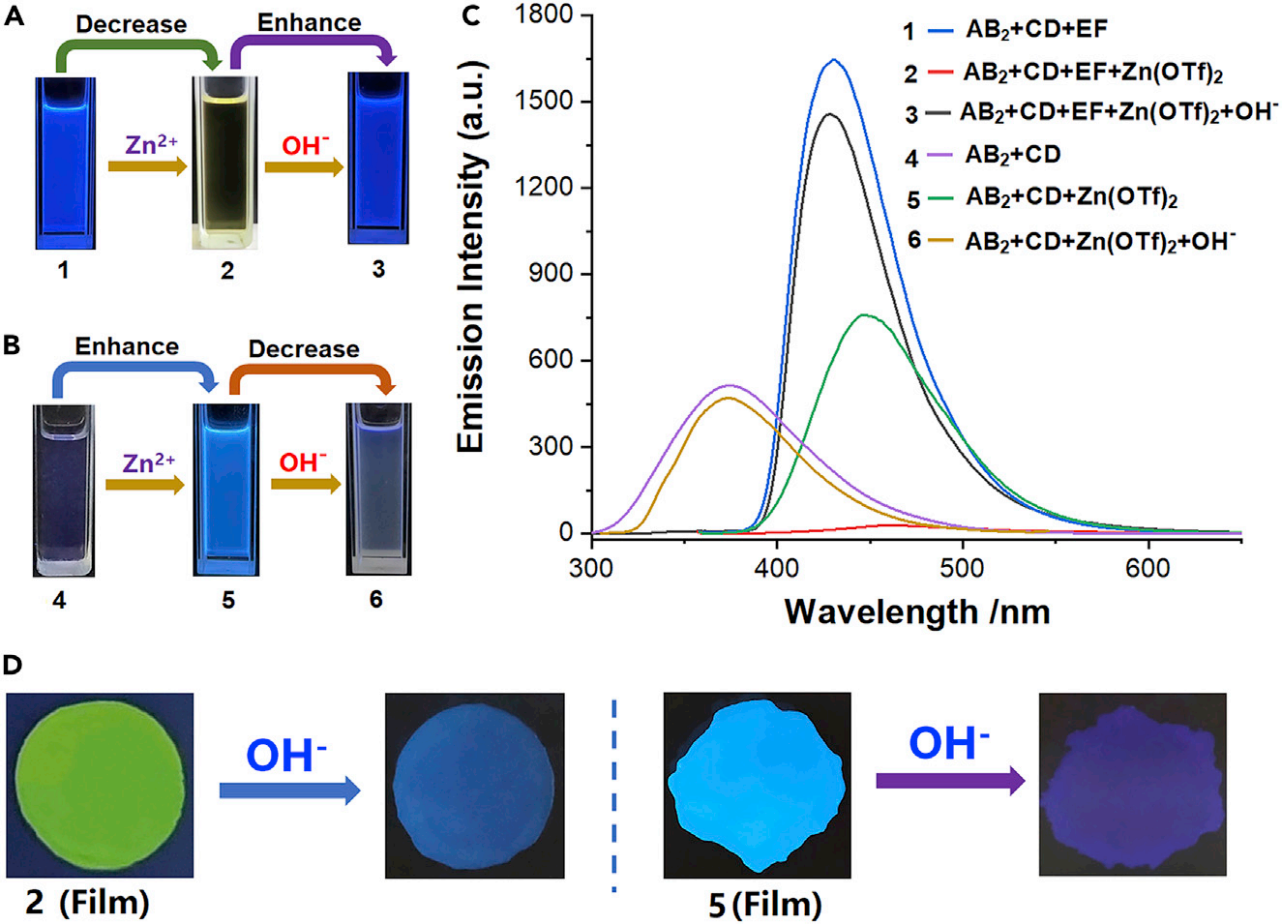
Photographs and Fluorescence emission spectra (A and B) Photographs of AB_2_+CD + EF, AB_2_+CD + EF + Zn(OTf)_2_, AB_2_+CD + EF + Zn(OTf)_2_ + TBAOH, AB_2_+CD, AB_2_+CD + Zn(OTf)_2_, and AB_2_+CD + Zn(OTf)_2_ + TBAOH in CDCl_3_−CD_3_COCD_3_ taken under 365 nm UV lamp irradiation. (C) Fluorescence emission spectra of the above solutions at 0.04 mM concentrations. (D) Photographs of the SP2 and SP3-based films and the TBAOH responsiveness of the films.

Besides the P5-TAPN, B21C-DAS host-guest interactions could be adjusted, the metal coordination tpy-Zn^2+^-tay may also be manipulated by adding OH^−^ anion. As shown in Figure 7A, the solution of AB_2_+CD + EF emitted strong blue fluorescence because of the anthracene chromophore on free tay group of EF. However, when Zn(OTf)_2_ was added to the above solution, the SP3 constructed by AB_2_+CD + EF + Zn(OTf)_2_ only gave off weak fluorescence owing to the formation of tpy-Zn^2+^-tay on the skeleton of SP3. When an organic base tetrabutylammonium hydroxide (TBAOH) was added into the solution of SP3, the fluorescence intensity of the solution almost recovered. The recovery of fluorescence emission could be explained as follows: When TBAOH was added into the solution, hydroxyl ion could form zinc hydroxide with zinc ion, which caused the destruction of tpy-Zn^2+^-tay coordination and the disassembly of SP3. The destruction of tpy-Zn^2+^-tay drove the coordinative tay into free tay, and the free tay group could emit strong blue fluorescence under the UV irradiation. The fluorescent spectra supported the inference (Figure 7C). Interesting, an opposite experimental phenomenon was observed in the SP2 system (Figure 7B), the solution of AB_2_+CD showed weak fluorescence emission. When Zn^2+^ ion was added to the AB_2_+CD solution, the AB_2_+CD + Zn(OTf)_2_ self-assembled into SP2 and a significant fluorescence enhancement was observed because of the formation of tpy-Zn^2+^-tpy coordination structure on the SP2 skeleton. However, When TBAOH was subsequently added into the solution of AB_2_+CD + Zn(OTf)_2_, the fluorescence emission significantly decreased owing to the dissociation of tpy-Zn^2+^-tpy coordination on the SP2 skeleton and the formation of zinc hydroxide. Furthermore, The SP2 and SP3-based films were prepared through spreading several drops of SP2 and SP3-containing solutions on glasses and then drying them in air. Similar fluorescence enhancement or quenching phenomena were observed after TBAOH were added to the above films (Figure 7D).

### Conclusions

In conclusion, we designed and synthesized three different monomers AB_2_, CD, and EF. The AB_2_ could self-assemble into supramolecular homopolymer SP1 based on P5-DAS host-guest interaction. When monomer CD and Zn(OTf)_2_ were added into the solution of SP1 solution, the SP1 was disassembled by competitive noncovalent interaction and simultaneously transformed into a new supramolecular copolymer SP2 based on the orthogonal noncovalent interaction of P5-TAPN and tpy-Zn^2+^-tpy. When monomer EF was further added into the solution of SP2, the SP2 further transformed into a new supramolecular copolymer SP3 with new sequence structure based on competitive self-sorting assembly. The multistep supramolecular assemblies were studied and verified by various techniques. The resulting three supramolecular polymers SP1, SP2, and SP3 showed different stimuli-responsiveness according to the types of noncovalent bonds. Various host–guest motifs and metal ligand pairs have been previously synthesized and offer choices to construct controllable supramolecular structures. Therefore, our assembly strategy may be adopted to construct tailored polymer sequences with structural variations and greater complexity by the combination of different noncovalent interactions. This research provides inspiration and possibilities for the design of supramolecular polymer with different advanced functions and sequence structures such as stimuli-responsiveness and self-repairing.

### Limitations of the study

This research provides inspiration for the design and preparation of supramolecular polymers with different sequence structures. However, the mechanical strength of supramolecular polymer materials is limited because of the dynamic reversibility of noncovalent reactions. Moreover, the synthesis of multiple monomers makes the supramolecular materials costly. Hence, functional supramolecular materials prepared by easier and cheaper methods should be exploited in future research.

## STAR★Methods

### Key resources table

**Table:** 

REAGENT or RESOURCE	SOURCE	IDENTIFIER
**Chemicals, peptides, and recombinant proteins**		

2-Acetyl-6-bromopyridine	Innochem	CAS:49669-13-8
NaOH	Innochem	CAS:1310-73-2
P-Anisaldehyde	Innochem	CAS:123-11-5
NH_3_·H_2_O	Greagent	CAS:1336-21-6
DCM	Greagent	CAS:75-09-2
Ethanol	Adamas	CAS:64-17-5
9-Anthraceneboronic acid	Innochem	CAS:100622-34-2
Tetrakis(triphenylphosphine)palladium(0)	Innochem	CAS:14221-01-3
Toluene	Greagent	CAS:108-88-3
Tert- butyl alcohol	Innochem	CAS:75-65-0
Na_2_CO_3_	Accela	CAS:497-19-8
CH_3_OH	Adamas	CAS:67-56-1
Hydrogen Bromide	Greagent	CAS:10035-10-6
Acetic Acid	Innochem	CAS:64-19-7
Na_2_SO_4_	Greagent	CAS:7757-82-6
Hexaethylene glycol	Innochem	CAS:2615-15-8
p-Toluenesulfonyl chloride	Innochem	CAS:98-59-9
Acetonitrile	Greagent	CAS:75-05-8
Triethylamine	Innochem	CAS:121-44-8
Tetrahydrofuran	Greagent	CAS:109-99-9
Petroleum Ether	Greagent	CAS:8032-32-4
Ethyl Acetate	Greagent	CAS:141-78-6
Methyl 3,4-dihydroxybenzoate	Local	CAS:2150-43-8
K_2_CO_3_	Greagent	CAS:584-08-7
Potassium fluoroborate	Innochem	CAS:14075-53-7
Tetrabutylammonium fluoride solution	Innochem	CAS:429-41-4
1,6-Dibromohexane	Innochem	CAS:629-03-8
N,N-Dimethylformamide	Greagent	CAS:68-12-2
Cesium Carbonate	Greagent	CAS:534-17-8
Paraformaldehyde	Innochem	CAS:30525-89-4
1,4-Dimethoxybenzene	Innochem	CAS:150-78-7
Boron trifluoride diethyl etherate	Innochem	CAS:109-63-7
Boron tribromide	Innochem	CAS:10294-33-4
Propargyl bromide	Innochem	CAS:106-96-7
Phloroglucinol	Innochem	CAS:108-73-6
1,2-Dibromoethane	Innochem	CAS:106-93-4
CuSO_4_·5H_2_O	Innochem	CAS:7758-99-8
Sodium ascorbate	Innochem	CAS:134-03-2
4-Hydroxybenzaldehyde	Innochem	CAS:123-08-0
Amylamine	Innochem	CAS:110-58-7
Hydrochloric Acid	Adamas	CAS:7647-01-0
Sodium borohydride	Innochem	CAS:16940-66-2
Silica gel	Greagent	CAS:63231-67-4
CDCl_3_	Innochem	CAS:865-49-6
Acetone-d_6_	Innochem	CAS:666-52-4
Zinc trifluoromethanesulfonate	Innochem	CAS:54010-75-2

**Software and algorithms**		

Origin 8	Origin Lab	https://www.originlab.com/

### Resource availability

#### Lead contact

Further information and requests for resources and reagents should be directed to and will be fulfilled by the lead contact, Hui Li (lh@jxust.edu.cn).

#### Material availability

Materials are available up on request.

### Experimental model and subject details

This study did not use experimental models typical in life sciences.

### Method details

#### General information

All starting materials were purchased from commercial sources and used without further purification. Compounds M1,^21^ M4,^52^ and monomer CD^45^ were synthesized using reported methodologies as described ahead. 200-300 mesh silica gel was adopted as column chromatography. ^1^H NMR, ^13^C NMR, 2D COSY, 2D NOESY and DOSY experiments were performed on a Bruker 400 MHz device or a Bruker 600 MHz device. Viscosity experiments were carried out using a ubbelohde viscometer (0.5 mm inner diameter) at 293 K. High-resolution MALDI-TOF mass spectra were recorded on a Bruker autoflex III mass spectrometer. Dynamic light scattering (DLS) experiments were performed on a Brookhaven BI-9000AT system (Brookhaven Instruments Corporation, USA), using a 200-mW polarized laser source (λ = 630 nm) and the solutions of the samples were filtered with a PTFE syringe filter before measurement. The UV−Vis experiments were performed on a Cary 60 UV-Vis spectrophotometer. The fluorescence spectra were performed on a Hitachi F-7000 spectrofluorometer. The XRD experiments were carried out on an Empyream instrument. The SEM samples were prepared at 35mM concentrations by casting droplet solutions of the samples on glass plates and the samples were dried for 12 hours. Gold coating were sputtered onto the samples and the samples were then observed on a JEOL 6390LV scanning electron microscopy instrument.

#### Synthesis of monomer AB_2_

A mixture of M1 (2.0g, 1.11mmol) and M2 (0.43g, 1.11mmol) in a solution of tetrahydrofuran and water (5:1, 100 mL) in the presence of CuSO_4_⋅5H_2_O (55.0mg, 0.22 mmol) with sodium ascorbate (105.0 mg, 0.54 mmol) was stirred at 60 °C for 12h. After the reaction mixture was cooled to ambient temperature, the solvent was evaporated under reduced pressure and the resulting residue was subjected to column chromatography (CH_2_Cl_2_/CH_3_OH= 40:1), to give AB_2_ (0.91g, 46%) as a white solid. ^1^H NMR (400 MHz, CD_3_COCD_3_, 298 K): ppm = 0.36 (m, 6H), 1.34(m, 2H), 3.08 (t, *J* = 6.8 Hz, 3H), 3.43 (s, 6H), 3.74 (m, 68H), 4.44 (t, *J* = 6.4 Hz, 2H), 5.02 (s, 4H), 5.23 (s, 2H), 5.68(s, 2H), 5.72 (s, 4H), 6.72(s, 2H), 6.93(m, 16H), 7.00(s, 2H), 7.09 (d, *J* = 8.4 Hz, 2H), 7.37(s, 2H), 7.50(s, 1H), 7.52 (d, *J* = 8.4 Hz, 2H), 8.03 (s, 1H), 8.22(s, 2H). ^13^C NMR (100 MHz, CDCl_3_): δ (ppm) = 159.33, 150.90, 150.58, 150.51, 149.26, 137.57, 131.71, 128.60, 128.34, 128.26, 128.18, 123.38, 115.47, 113.83, 113.75, 113.62, 113.45, 113.40, 113.34, 62.09, 61.50, 55.01, 54.97, 52.99, 52.84, 51.11, 47.92, 27.51, 25.44, 21.01, 13.14. MALDI-TOF-MS (C_118_H_131_F_6_N_10_O_21_P): *m/z* calcd for [M – PF_6_^**-**^]^**+**^= 2024.9518, found =2024.9567, error 2.4 ppm. See Scheme S1, Figures S35–S37.

#### Synthesis of monomer AE

A solution of M3 (2.00 g, 1.48 mmol), M4 (0.83 g, 1.48 mmol), Cs_2_CO_3_ (1.45 g, 4.5mmol) in DMF (50 mL) was stirred for 14 hat 82 °C. After the reaction mixture was cooled to ambient temperature, the organic solvent was evaporated under reduced pressure, and the residue was partitioned between dichloromethane (50 mL) and water (50 mL). The aqueous layer was further washed with dichloromethane (2 × 50 mL). The organic phases were combined and dried by anhydrous Na_2_SO_4_. After the solvent was removed, the resulting residue was subjected to column chromatography (dichloromethane/methanol=70:1), to give AE (1.28g, 74%) as a white solid. ^1^H NMR (400 MHz, CDCl_3_): *δ* (ppm) 8.97 (d, *J* = 8.0 Hz, 2H), 8.69 (s, 2H), 8.61 (s, 2H), 8.19 (t, *J* = 8.0 Hz, 2H), 8.10 (d, *J* = 8.0 Hz, 2H), 7.77 (d, *J* = 8.0 Hz, 4H), 7.56–7.65 (m, 5H), 7.49–7.53 (m, 5H), 7.35–7.42 (m, 4H), 6.84 (d, *J* = 8.0 Hz, 1H), 6.78 (d, *J* = 8.0 Hz, 2H), 4.27 (t, *J* = 6.6 Hz, 2H), 4.16–4.21 (m, 4H), 3.89–3.98(m, 4H), 3.87(t, *J* = 4.0 Hz, 2H), 3.77–3.83(m, 4H), 3.71–3.76(m, 4H), 3.63–3.70(m, 8H), 1.72–1.79 (m, 4H), 1.43–1.47(m, 4H). ^13^C NMR (100 MHz, CDCl_3_): δ (ppm) = 166.4, 159.8, 157.6, 156.8, 155.9, 152.8, 148.3, 137.1, 135.6, 131.5, 130.2, 128.5, 127.5, 127.0, 126.4, 125.9, 125.2, 123.9, 120.0, 118.9, 114.6, 112.3, 71.3, 71.2, 71.1, 70.9, 70.7, 69.7, 69.5, 69.0, 67.7, 64.8, 29.0, 28,7, 25.8, 25.7. MALDI-TOF-MS (C_74_H_69_N_3_O_10_): m/z calcd for [M+H]^+^ =1160.5056, found =1160.5066, error 1.0 ppm. See Scheme S2, Figures S38–S40.

#### The study of binding constants

##### Tpy-Zn2+-tay binding constant

###### Method A: Job plot of UV-Vis titration

To determine the association constant of tpy-Zn^2+^-tay, the UV-Vis experiment (Job plot method) was conducted according to the literature method.^53^ Model compounds 3 and 4 were chosen as the ligands. A series of samples were prepared and the total molar concentration of ligands ($\frac{\left[3\right]+\left[4\right]}{2}$) and zinc ion was maintained at 2×10^-5^M in each sample and only the ratios of zinc ion to ligands were altered. The job plot was conducted by varying the mole fractions of the ligands ($\frac{\left[3\right]+\left[4\right]}{2}$) and zinc ion. The concentration: $\frac{\left[3\right]+\left[4\right]}{2}$ + [Zn(OTf)_2_] = 2×10^-5^M. The absorbance intensity at 412 nm was plotted (Figure S18) against the mole fraction of Zn(OTf)_2_. The Job plot (Figure S18) indicates a 1:1:1 binding among Zn^2+^, 3 and 4.

Furthermore, the data of job plot were divided into two groups around X_m_= 0.5. When X_m_ ≤ 0.5, the fitting equation is A = 0.1766X_m_ + 0.01223. When X_m_≥ 0.5, the fitting equation is A = -0.2015X_m_ + 0.20071. The intersection point of the two fitting curves is taken (X_m_=0.5075, A=0.1031), and the experimental value is X_m_=0.5000, A'= 0.0991. The dissociation degree of complex [Zn**34**](OTf)_2_ was calculated from Equation 1. According to the formula, the dissociation degree(*α)* of complex [Zn**34**](OTf)_2_ was calculated to be 0.038.

(Equation 1) $$\alpha =\left(A-A^{\prime }\right)/A\text{,}$$

The binding constant *K* was then calculated to be 6.68×10^7^ M^−1^ based on Equation 2.

(Equation 2) $$K=\frac{{\left[Zn34\right]\left(OTf\right)}_{2}}{{\left[4\right]\;\left[Zn3\right]\left(OTf\right)}_{2})}=\frac{1-\alpha }{{C\alpha }^{2}}$$

Where *C* is the total concentration of the complex [Zn**34**](OTf)_2_ and *α* is the degree of dissociation of complex [Zn**34**](OTf)_2_ when X_m_ value is 0.5, with the hypothesis that the ligands and zinc ion only form the complex [Zn**34**](OTf)_2_. The *C* is 1×10^-5^ M and the *α* is 0.038 when X_m_ is 0.5.

###### **Method B:**Isothermal titration calorimetry (ITC)

The binding constant tpy-Zn^2+^-tay was also determined in CDCl_3_-CD_3_COCD_3_ (3:1, v/v) by using isothermal titration calorimetry (ITC). A representative calorimetric titration curve was shown in Figure S19. Based on the obtained ITC data, the association constant (*K*) of tpy-Zn^2+^-tay was estimated to be (5.85±3.03)×10^7^ M^−1^.

The binding constant of tpy-Zn2+-tay measured by job plot method is consistent with the measured value of isothermal titration calorimetry.

##### Tpy-Zn^2+^-tpy binding constant

###### Method A: Job plot of UV-vis titration

To determine the association constant of tpy-Zn^2+^-tpy, the UV-Vis experiment (Job plot method) was conducted according to the literature method.^53^ Model compounds 3 was chosen as the ligand. A series of samples were prepared and the total molar concentration of ligand 3 and zinc ion was maintained at 2×10^-5^M in each sample and only the ratios of zinc ion to ligand were altered. The job plot was conducted by varying the mole fractions of the ligand 3 and zinc ion. The concentration: 3+ [Zn(OTf)_2_] = 2×10^-5^M. The absorbance intensity at 348 nm was plotted (Figure S20) against the mole fraction of Zn(OTf)_2_. The Job plot indicates a 1:2 binding ratio between Zn^2+^ and 3.

Furthermore, the data of job plot were divided into two groups around X_m_= 0.5. When X_m_ ≤ 0.5, the fitting equation is A = 1.57516X_m_ + 0.02735. When X_m_≥ 0.5, the fitting equation is A = -0.81109X_m_ + 0.819. The intersection point of the two fitting curves is taken (X_m_=0.331, A=0.587), and the experimental value is X_m_=0.333, A'=0.524. The dissociation degree of complex [Zn**3**_**2**_](OTf)_2_ was calculated from Equation 3. According to the formula, the dissociation degree(*α)* of complex [Zn**3**_**2**_](OTf)_2_ was calculated to be 0.107.

(Equation 3) $$\alpha =\left(A-A^{\prime }\right)/A\text{,}$$

The binding constant *K* was calculated to be 7.76×10^6^ M^−1^ based on Equation 4.

(Equation 4) $$K=\frac{{\left[Zn3_{2}\right]\left(OTf\right)}_{2}}{{\left[3\right]\;\left[Zn3\right]\left(OTf\right)}_{2})}=\frac{1-\alpha }{{C\alpha }^{2}}$$

###### Method B: Isothermal titration calorimetry (ITC)

The binding constant tpy-Zn^2+^-tpy was also determined in CDCl_3_-CD_3_COCD_3_ (3:1, v/v) by isothermal titration calorimetry (ITC) method. A representative calorimetric titration curve was shown in Figure S21. Based on the obtained ITC data, the association constant (*K*) of tpy-Zn^2+^-tpy was estimated to be (1.00±0.32)×10^7^ M^−1^.

##### P5-DAS binding constant

To investigate the association constant of pillar[5]arene/dialkylammonium salt (P5-DAS) between pillar[5]arene moiety of AB_2_ and dialkylammonium salt moiety of AB_2_ in CDCl_3_, ^1^H NMR titrations were performed with a constant concentration of model compound 6 (2.00 mM) and varying the concentrations of model compound 1 in the range of 0.25–6.0 mM. By a non-linear curve-fitting method,^39^ the association constant (*K*a) of P5-DAS was estimated to be (432 ±7) M^−1^ (Figures S22 and S23).

The non-linear curve-fittings were based on the equation:

(Equation 5) $$\Delta \delta =(\Delta \delta \infty /{\left[G\right]}_{0})\;(\;{0.5{\left[H\right]}_{0}+0.5({\left[G\right]}_{0}+1/Ka)-(0.5(\;{\left[H\right]}_{0}^{2}+(2{\left[H\right]}_{0}(1/Ka-{\left[G\right]}_{0}))+{\left(1/Ka+{\left[G\right]}_{0}\right)}^{2})}^{0.5}))$$

Where *Δδ* is the chemical shift change of C_14_ on model compound 6 at [H]_0_, *Δδ*∞ is the chemical shift change of C_14_ when the compound 6 is completely complexed, [G]_0_ is the fixed initial concentration of the guest molecule 6, and [H]_0_ is the varying concentrations of host molecule 1.

##### P5-TAPN binding constant

We used model compound 1 and 2 to determine the binding constant *K*_*a*_ of the P5-TAPN in CDCl_3_-CD_3_COCD_3_ (3:1, v/v, 298K) according to the reported method.^42^ Because P5-TAPN was a slow exchange interaction, the *K*_*a*_ could be calculated from integrations of complexed and uncomplexed peaks in ^1^H NMR spectrum. The experiment was performed at 2.00 mM CDCl_3_-CD_3_COCD_3_ solution. Using the reference method, *K*_a_ value was calculated as below:[(3.78/4.78) × 2 × 10^−3^]/[(1–3.78/4.78) × 2 × 10^−3^]^2^ = 9.03×10^3^ M^−1^ (Figure S24).

##### B21C-DAS binding constant

The B21C-DAS host-guest interaction is a slow exchanging interaction. We use model compound 5 and 6 to determine the binding constant *K*_*a*_ of the B21C-DAS in CDCl_3_-CD_3_COCD_3_ according to the reported method.^52^ Because B21C-DAS is a slow exchange interaction, the *K*_*a*_ could be calculated from integrations of complexed and uncomplexed peaks in ^1^H NMR spectrum. The experiment was performed at 5.00 mM CDCl_3_-CD_3_COCD_3_ solution. Using the reference method, *K*_a_ value was calculated as below:[(1.48/2.48) × 5 × 10^−3^]/[(1–1.48/2.48) × 5 × 10^−3^]^2^ = 733 M^−1^ (Figure S25).

## Data Availability

• Data reported in this article will be shared by the lead contact on request. • There is no dataset or code associated with this work. • Any additional information required to reanalyse the data reported in this study is available from the lead contact upon request.

## Supplemental information

Document S1. Figures S1–S40 and Schemes S1 and S2
