# Supplementary material for: Multistep sequence-controlled supramolecular polymerization by the combination of multiple self-assembly motifs
Source: iScience. 2023 Jan 23;26(2):106023. doi: 10.1016/j.isci.2023.106023 (PMC9932128; doi:10.1016/j.isci.2023.106023)
Supplement: Document S1. Figures S1–S40 and Schemes S1 and S2 [file mmc1.pdf]

iScience, Volume *26*

Supplemental information

Multistep sequence-controlled supramolecular polymerization by the combination of multiple self-assembly motifs

Hui Li, Shenghui Rao, Ying Yang, Fenfen Xu, Zhe Huang, Xiaohui Huang, Zhu Zhu, Shengyong Liu, Zhelin Zhang, and Wei Tian

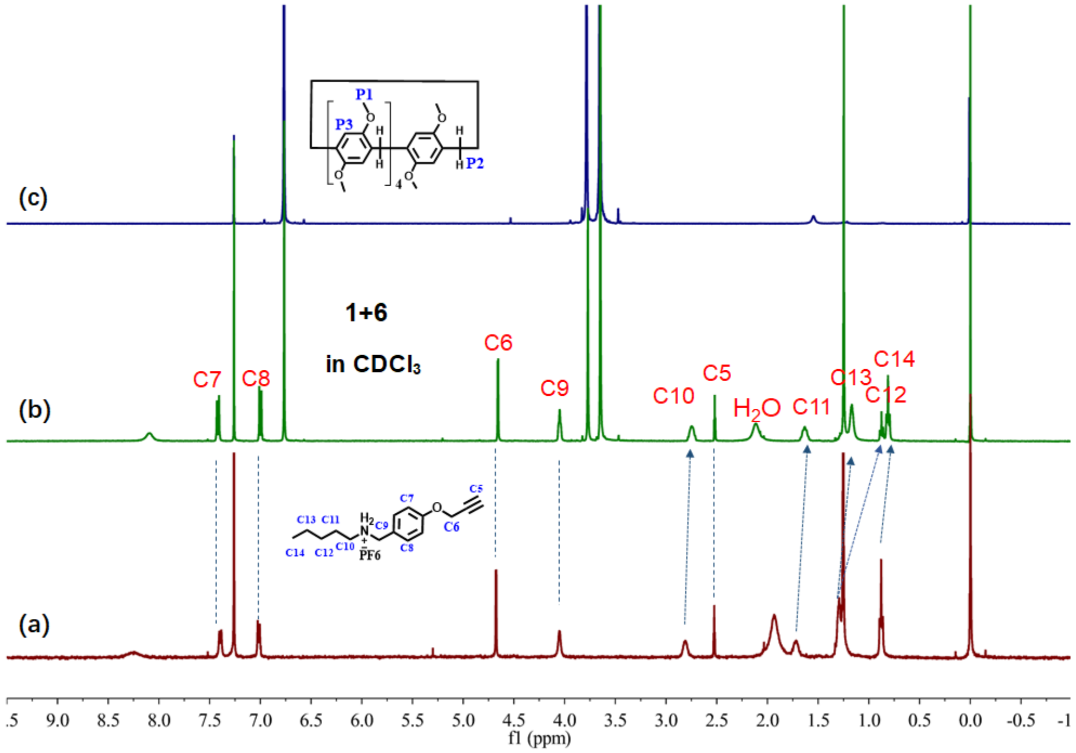

Fig. S1 ^{1}H NMR spectra (400 MHz, chloroform-d_3, 298 K) of (a) **6**, (b) an equimolar solution of **1** and **6**, (c) **1.** Related to Figure 1.

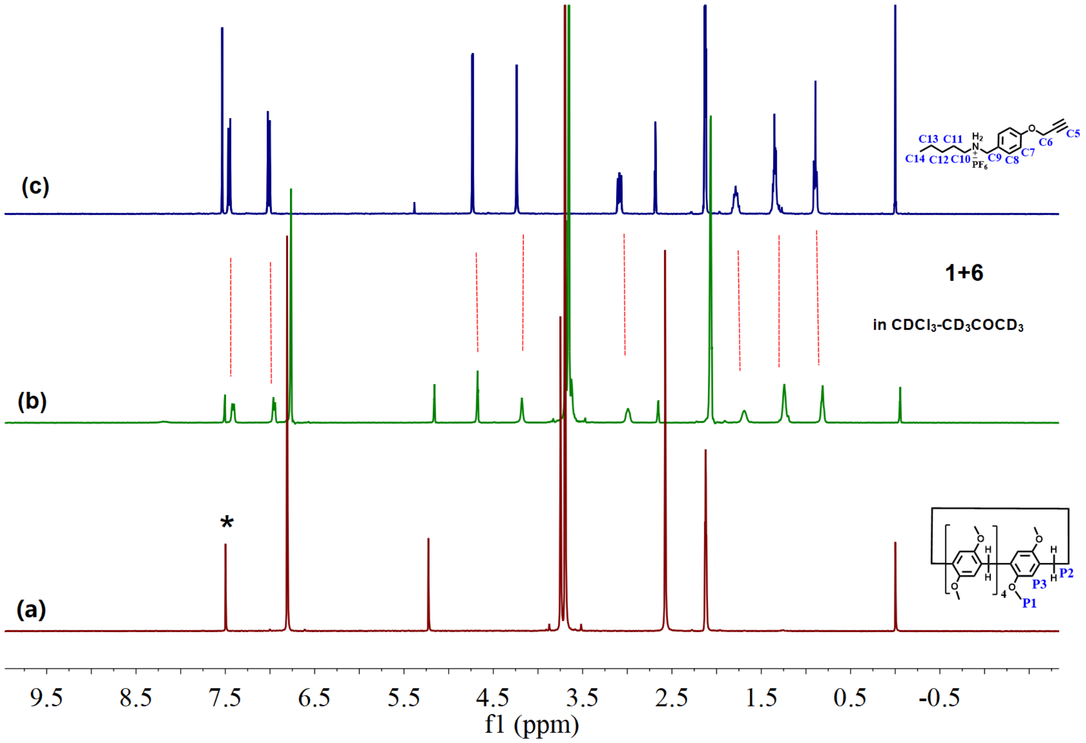

Fig. S2 ^{1}H NMR spectra (400 MHz, chloroform-d_3/acetone-d_6(3/1, v/v), 298 K) of (a) **1**, (b) an equimolar solution of **1** and **6**, (c) **6.** Related to Figure 1.

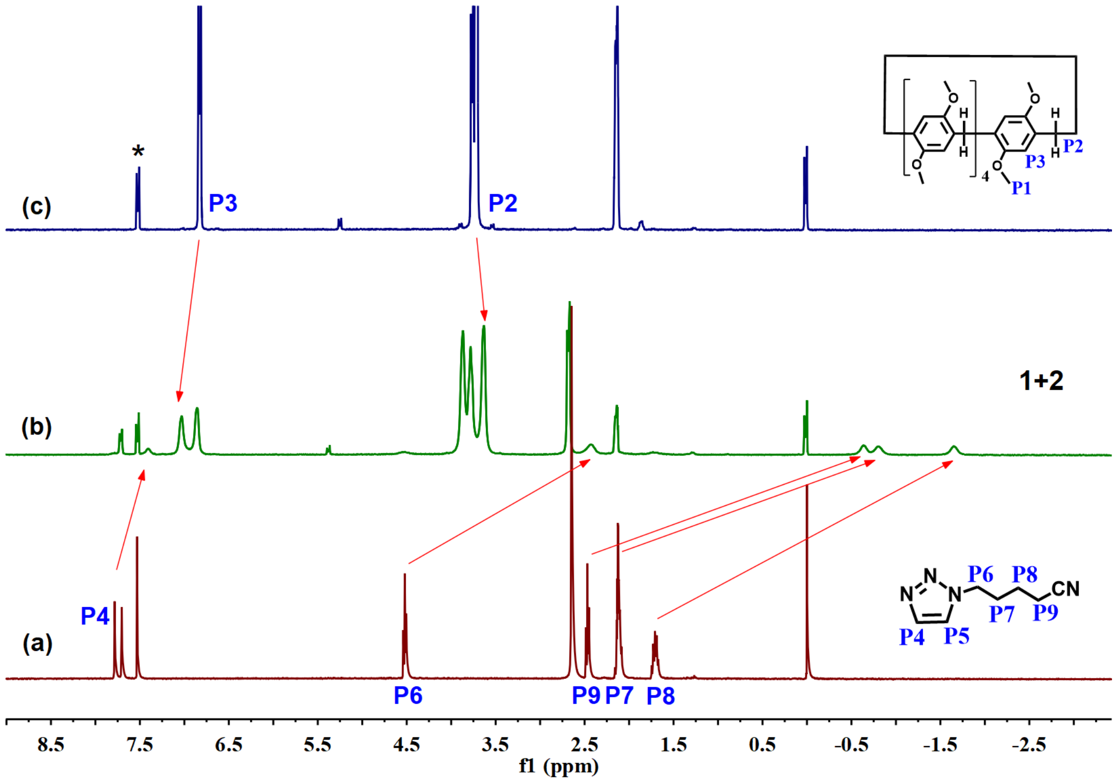

Fig. S3 ^{1}H NMR spectra (400 MHz, chloroform-d_3/acetone-d_6(3/1, *v*/*v*), 298 K) of (a) **2**, (b) an equimolar solution of **1** and **2**, (c) **1.** Related to Figure 1.

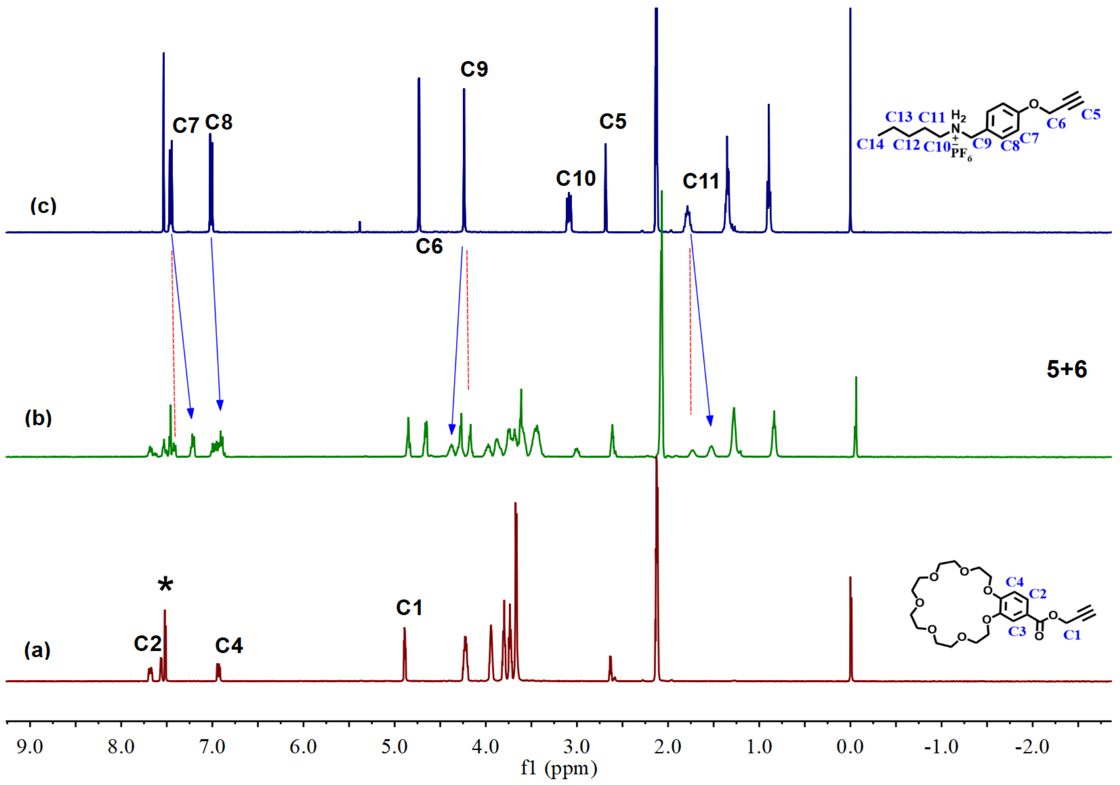

Fig. S4 ^{1}H NMR spectra (400 MHz, chloroform-d_3/acetone-d_6(3/1, *v*/*v*), 298 K) of (a) **5**, (b) an equimolar solution of **5** and **6**, (c) **6.** Related to Figure 1.

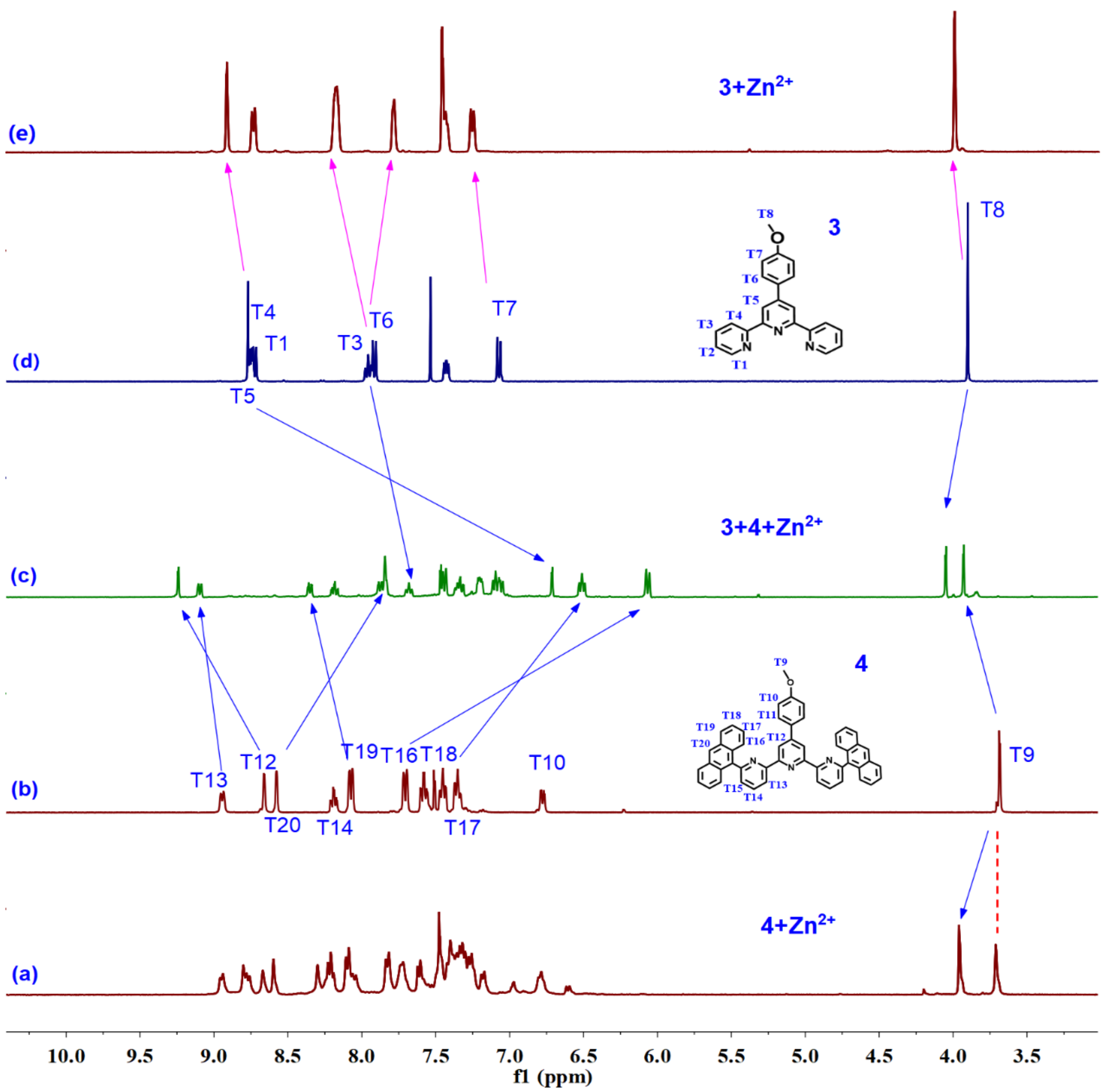

Fig. S5 ^{1}H NMR spectra (400 MHz, chloroform-*d3*/acetone -*d3*=3/1, v/v, 298 K) of (a) 2:1 molar ratio of 4+Zn(OTf)$_2$, (b) 4, (c) 1:1:1 molar ratio of 3+4+Zn(OTf)$_2$, (d) 3, (e) 2:1 molar ratio of 3+Zn(OTf)$_2$. Related to Figure 1.

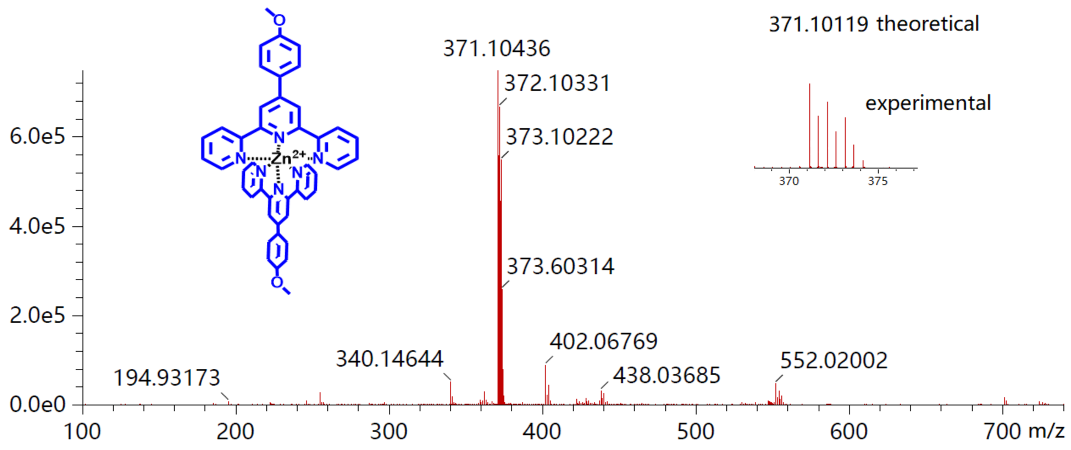

Fig. S6 ESI-MS spectra of $[Zn3_2]^{2+}$ at 0.5mM concentration in $CHCl_3$-CH_3COCH_3(3:1, v/v). Related to Figure 1.

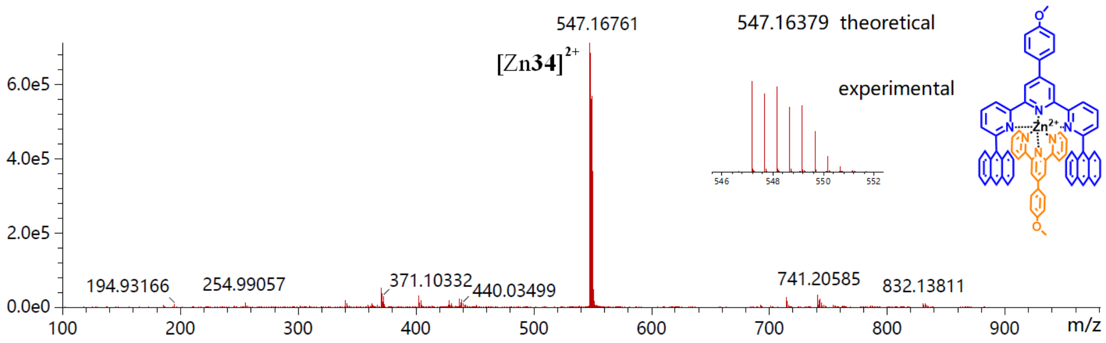

Fig. S7 ESI-MS spectra of $[Zn34]^{2+}$ at 0.5mM concentration in $CHCl_3$-CH_3COCH_3(3:1, v/v). Related to Figure 1.

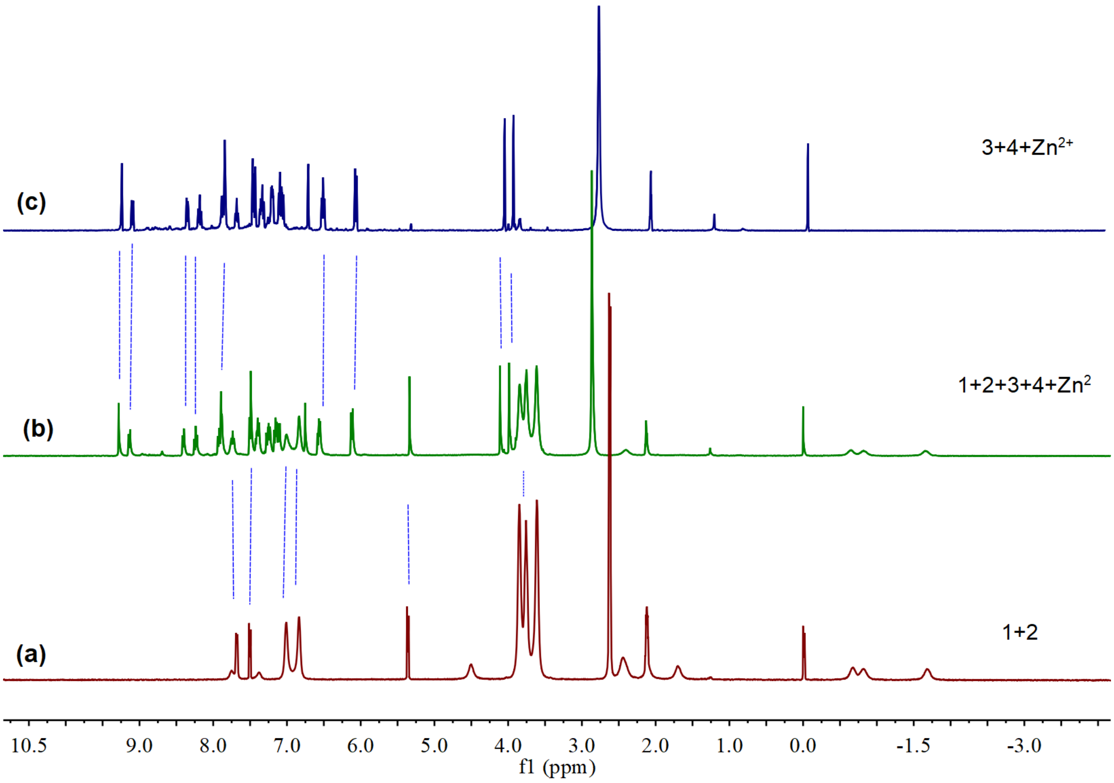

Fig. S8 ^{1}H NMR spectra (400 MHz, chloroform-d_3/acetone-d_6(3/1, *v*/*v*), 298 K) of (a) **1+2**, (b) **1+2+3+4+Zn(OTf)₂**, (c) **3+4+Zn(OTf)₂**. Related to Figure 1.

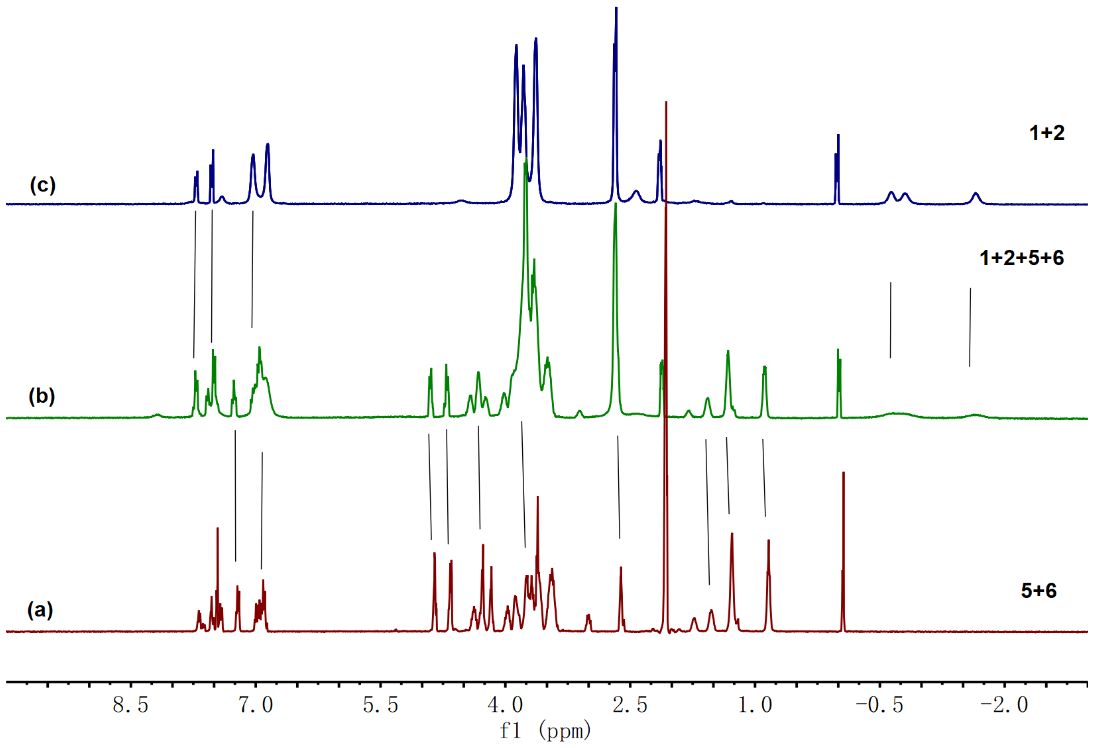

Fig. S9 ^{1}H NMR spectra (400 MHz, chloroform-d_3/acetone-d_6(3/1, *v*/*v*), 298 K) of (a) **5+6**, (b) **1+2+5+6**, (c) **1+2**. Related to Figure 1.

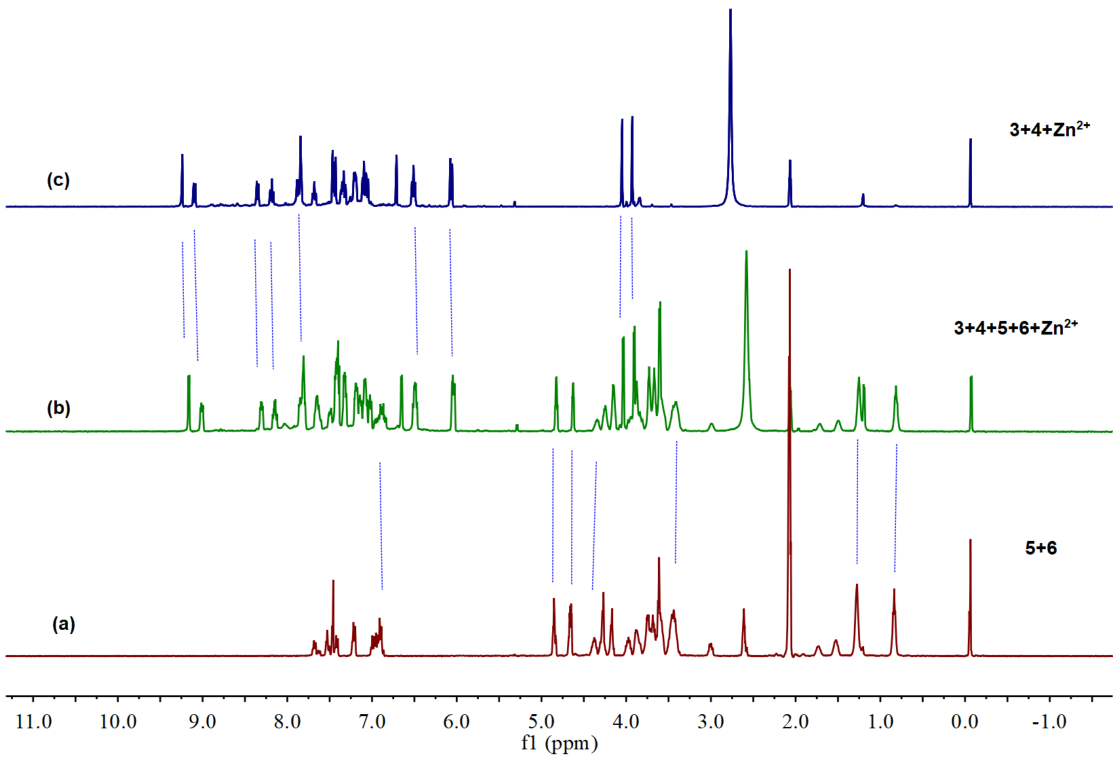

Fig. S10 ^{1}H NMR spectra (400 MHz, chloroform-d_3/acetone-d_6(3/1, *v*/*v*), 298 K) of (a) **5+6**, (b) **3+4+5+6+Zn(OTf)$_2$**, (c) **3+4+Zn(OTf)$_2$**. Related to Figure 1.

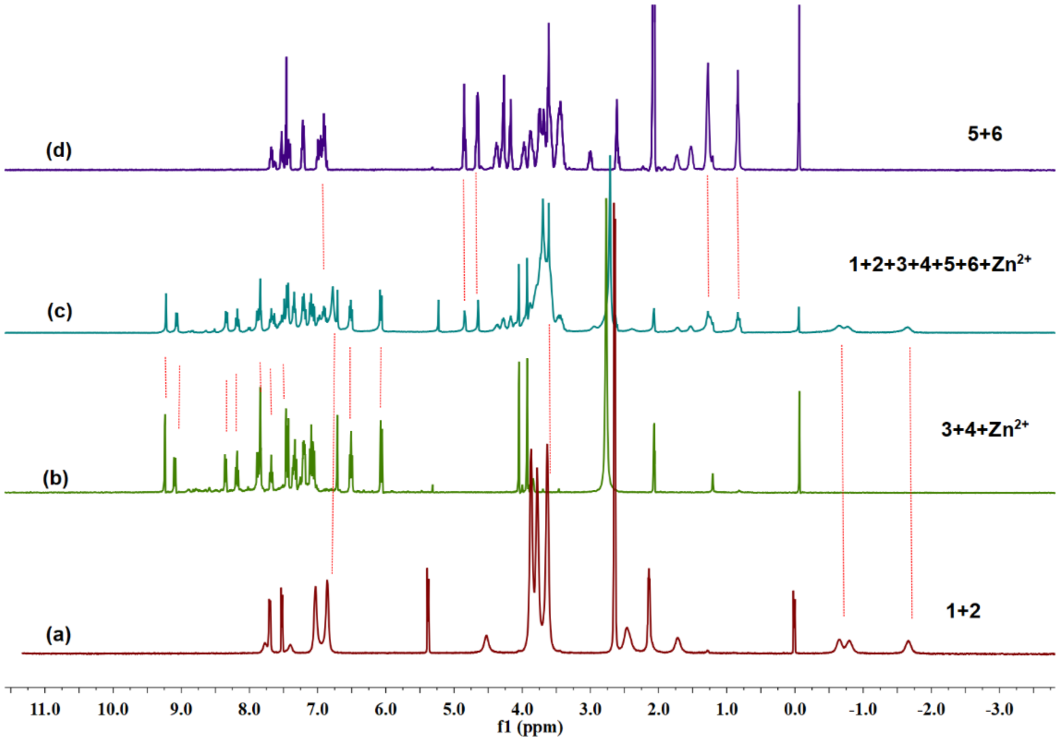

Fig. S11 ^{1}H NMR spectra (400 MHz, chloroform-d_3/acetone-d_6(3/1, *v*/*v*), 298 K) of (a) **1+2**, (b) **3+4+**$\mathbf{Zn(OTf)_2}$, (c) **1+2+3+4+5+6+**$\mathbf{Zn(OTf)_2}$**, (d)5+6**. Related to Figure 1.

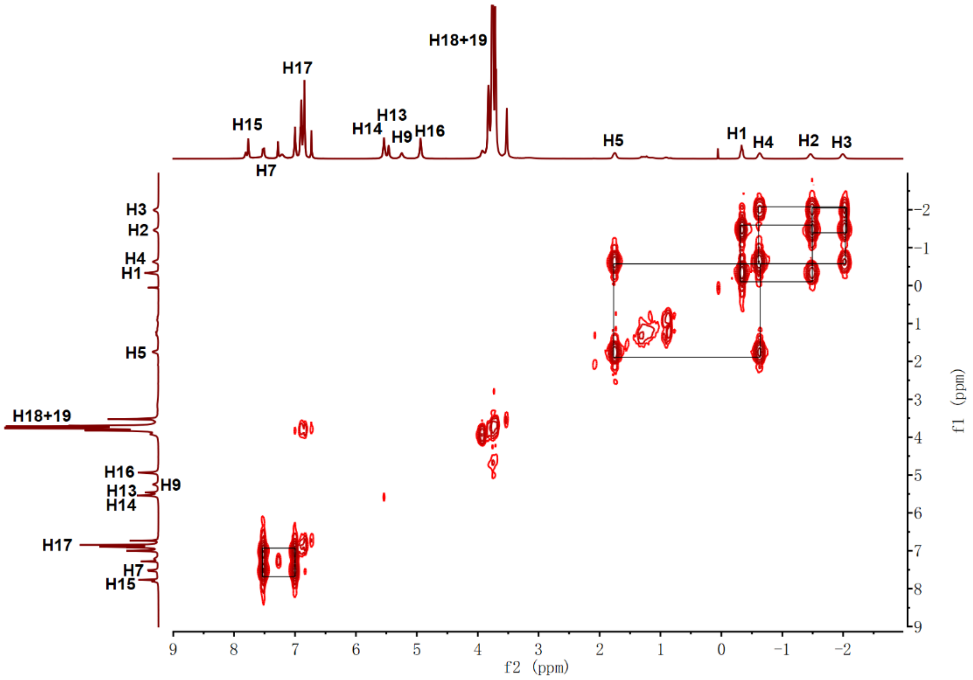

Fig. S12 ^{1}H-^{1}H COSY NMR spectrum (400 MHz, $CDCl_3$, 298 K, 40mM) of AB_2. The strong correlations between the protons H1 and H2, between the protons H2 and H3, and between H3 and H4 on AB_2 were observed, the correlations between H4 and H5 was also observed at the same time. By means of the ^{1}H–^{1}H COSY experiment, the complex ^{1}H NMR spectrum of AB_2 was identified. Related to Figures 1 and 2.

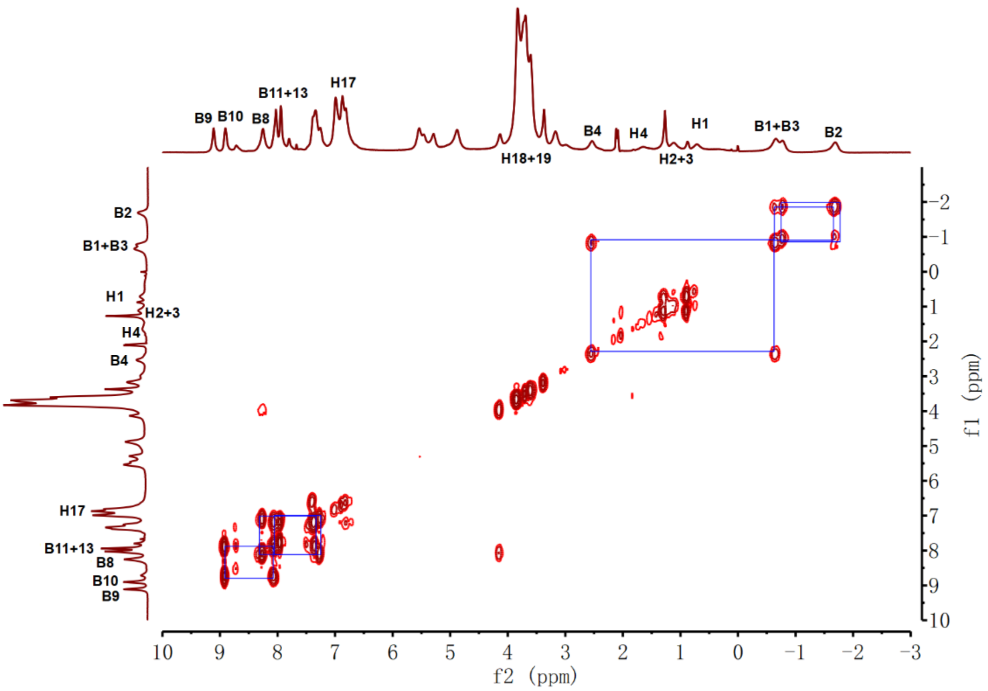

Fig. S13 ^{1}H-^{1}H COSY NMR spectrum (400 MHz, $CDCl_3$-CD_3COCD_3 = 3/1, v/v, 298 K, 40mM) of AB_2+CD+Zn$(OTf)_2$. The strong correlations between the protons B1 and B2 and between B3 and B4 on CD were observed, the correlations between H4 and H5 was also observed at the same time. By means of the ^{1}H–^{1}H COSY experiment, the complex ^{1}H NMR spectrum of AB_2+CD+Zn$(OTf)_2$ was identified. Related to Figures 1 and 2.

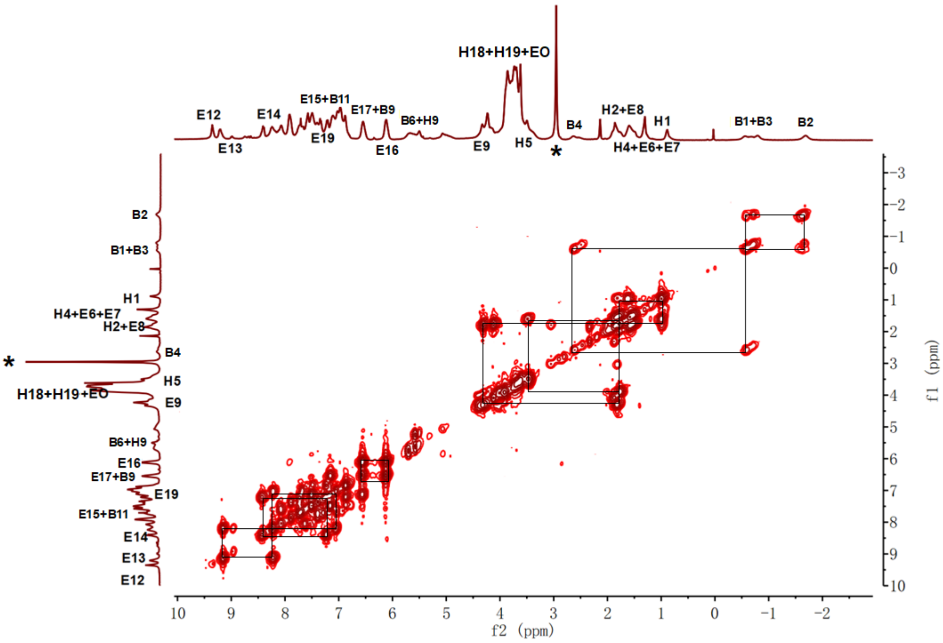

Fig. S14 ^{1}H-^{1}H COSY NMR spectrum (400 MHz, $CDCl_3$-CD_3COCD_3 = 3/1, v/v, 298 K, 40mM) of AB_2+CD+EF+Zn$(OTf)_2$. The strong correlations between the protons B1 and B2 and between B3 and B4 on CD were observed, the correlations between H4 and H5 on AB2, E8 and E9 on EF were also observed at the same time. By means of the ^{1}H–^{1}H COSY experiment, the complex ^{1}H NMR spectrum of AB_2+CD+EF+Zn$(OTf)_2$ was identified. Related to Figures 1 and 2.

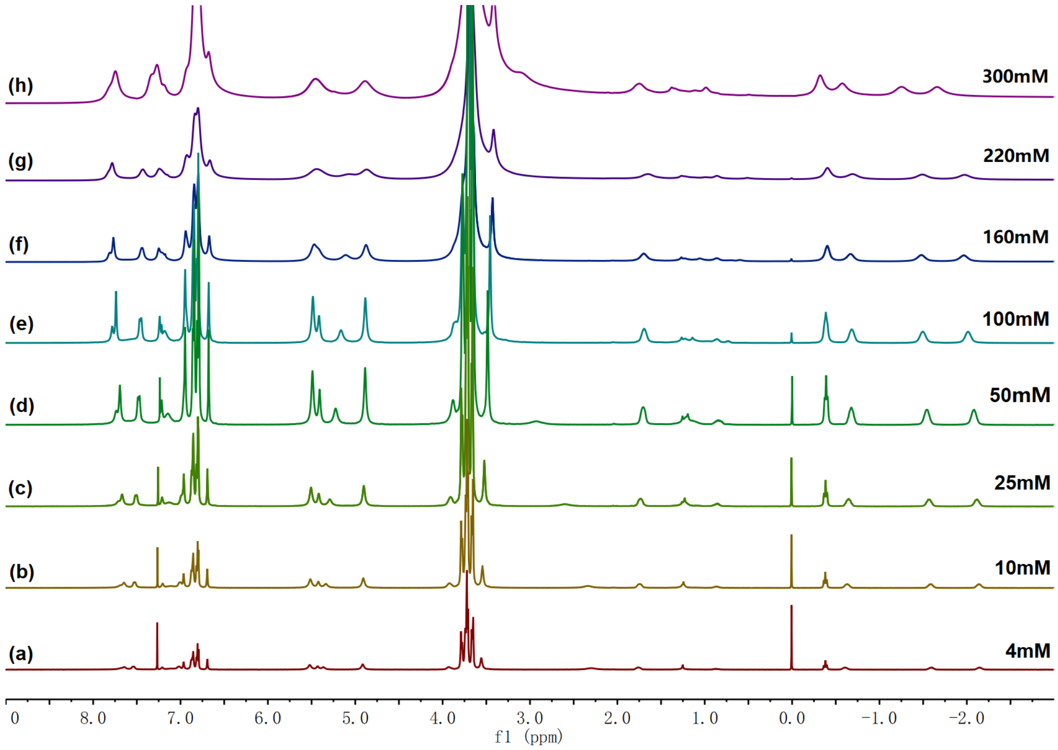

Fig. S15 ^{1}H NMR spectra (400 MHz, $CDCl_3$, v/v, 298 K) of AB_2 at different concentrations (a) 4 mM, (b) 10 mM, (c) 25 mM, (d) 50 mM, (e) 100 mM, (f) 160 mM, (g) 220mM, (h) 300mM. Related to Figures 1 and 2.

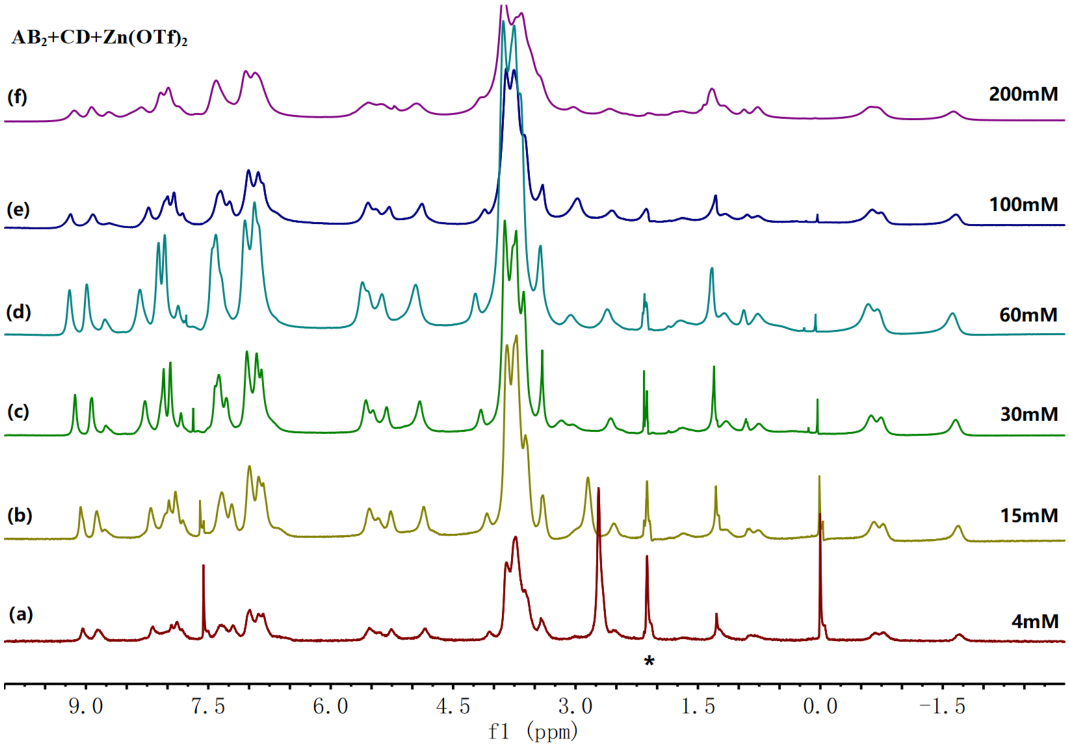

Fig. S16 ^{1}H NMR spectra (400 MHz, $CDCl_3$-CD_3COCD_3 = 3/1, v/v, 298 K) of AB_2+CD+$Zn(OTf)_2$ at different concentrations (a) 4 mM, (b) 15 mM, (c) 30 mM, (d) 60 mM, (e) 100 mM, (f) 200 mM. Related to Figures 1 and 2.

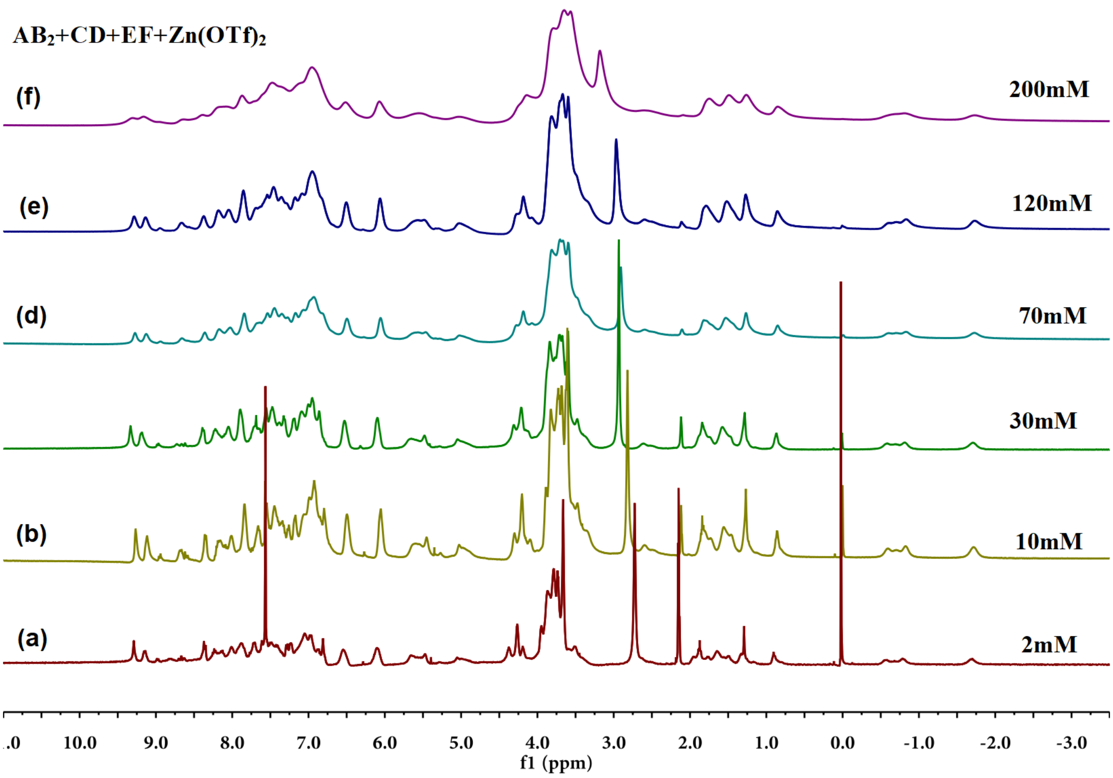

Fig. S17 ^{1}H NMR spectra (400 MHz, $CDCl_3$-CD_3COCD_3 = 3/1, v/v, 298 K) of AB_2+CD+EF+$Zn(OTf)_2$ at different concentrations (a) 2 mM, (b) 10 mM, (c) 30 mM, (d) 70 mM, (e) 120 mM, (f) 200 mM. Related to Figures 1 and 2.

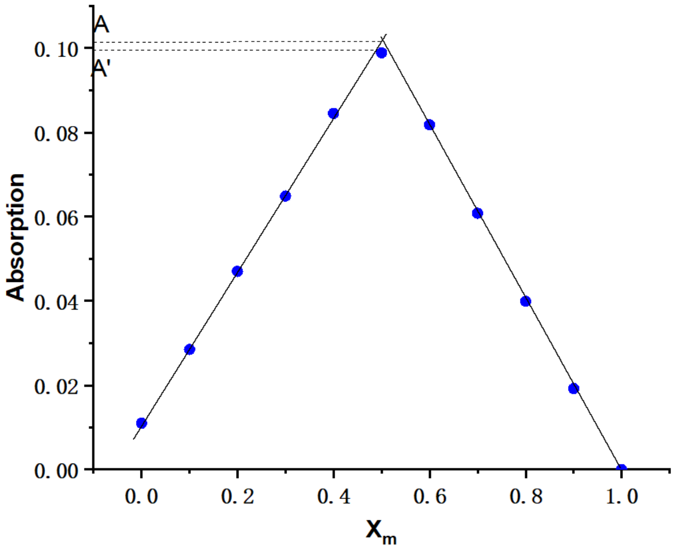

Fig. S18 Job plot of the complex formed among zinc ion, 3 (ligand) and 4 (ligand) showing a 1:1:1 stoichiometry by plotting the absorbance intensity at 412 nm against the mole fraction of zinc ion. Concentration: [**3**]=[**4**], $\frac{[3]+[4]}{2}$ + [$Zn(OTf)_2$] = 2×10^{-5}M. (chloroform versus acetone=3:1, v/v, 298K). Related to Figures 1 and 2. Related to STAR Methods and Figure 3.

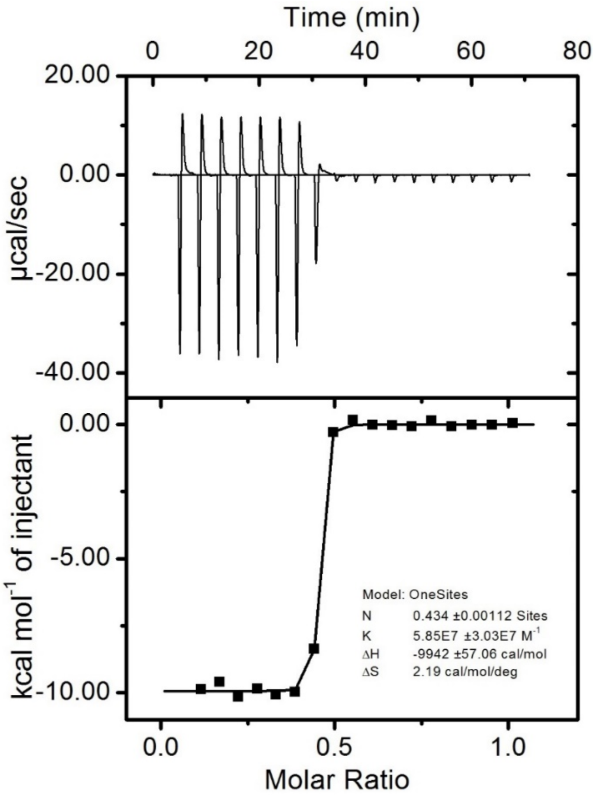

Fig. S19. Fitting the ITC data for titration experiments of $Zn(OTf)_2$ (3.75 mM) into the solution of 3+4 (0.5 mM) with a one-sites binding model ($CHCl_3$-CH_3COCH_3, 3:1, v/v). Related to STAR Methods and Figure 3.

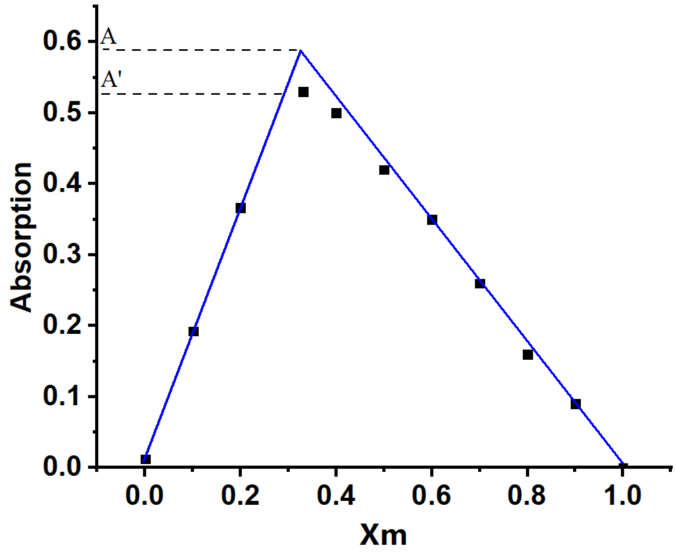

Fig. S20 Job plot of the complex formed between zinc ion, 3 (ligand) showed a 1:2 stoichiometry by plotting the absorbance intensity at 348 nm against the mole fraction of zinc ion. Concentration: [**3**] + [$Zn(OTf)_2$] = 2×10^{-5}M. (chloroform versus acetone=3:1, v/v, 298K). Related to STAR Methods and Figure 3.

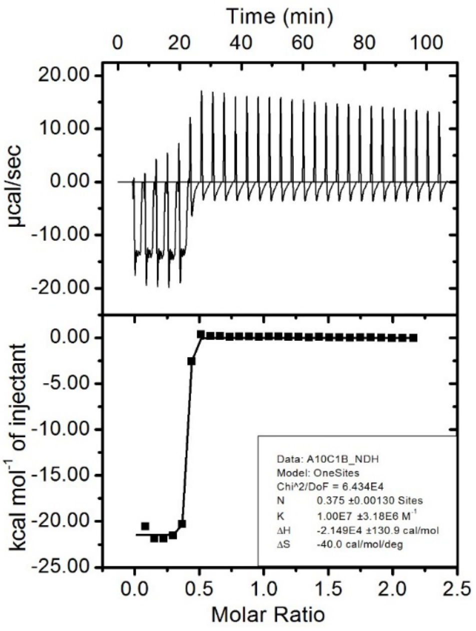

Fig. S21 Fitting the ITC data for titration experiments of $Zn(OTf)_2$ (5.00 mM) into the solution of 3 (0.5 mM) with a one-sites binding model ($CHCl_3$-CH_3COCH_3, 3:1, v/v). Related to STAR Methods and Figure 3.

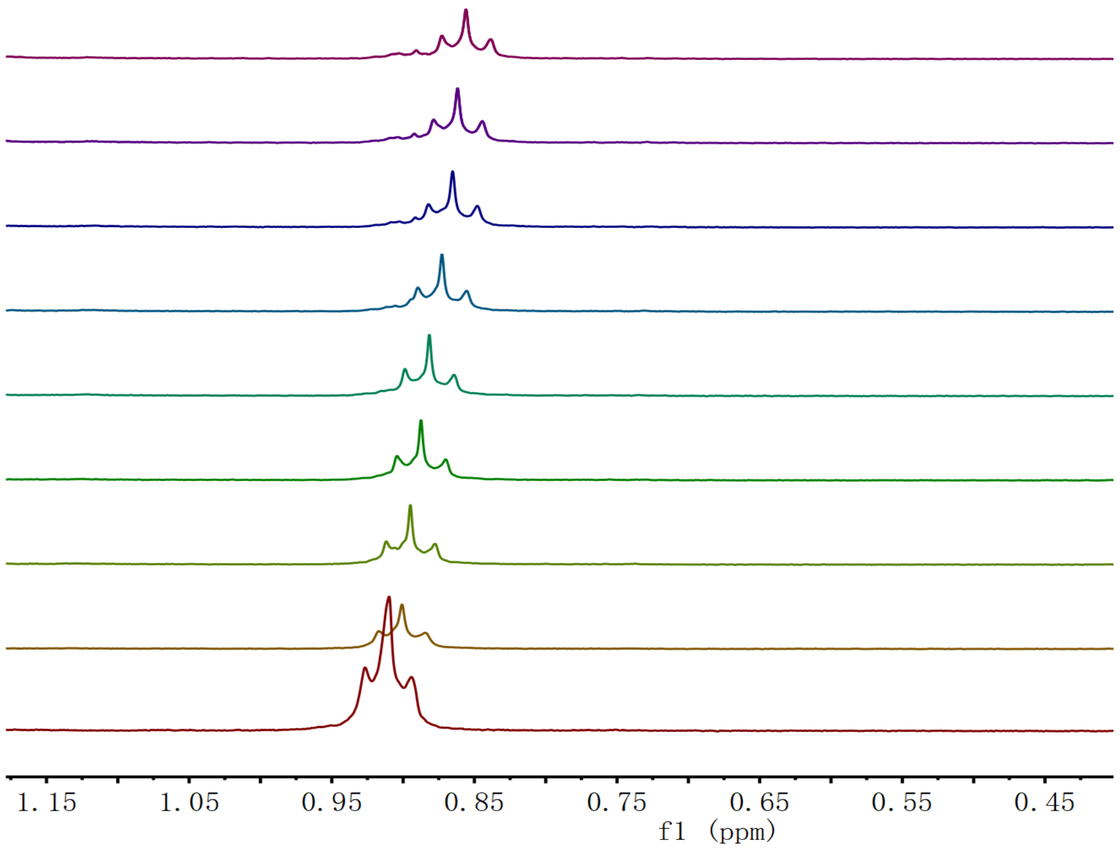

Fig. S22 Partial ^{1}H NMR titration spectra (400 MHz, $CDCl_3$, 298K) of 2.00 mM **6** solution with (a) 0, (b)0.5mM,

(c) 1mM, (d) 1.5mM, (e) 2mM, (f) 3mM, (g) 4mM, (h) 5mM, and (i) 6mM of **1**. Related to STAR Methods and Figure 3.

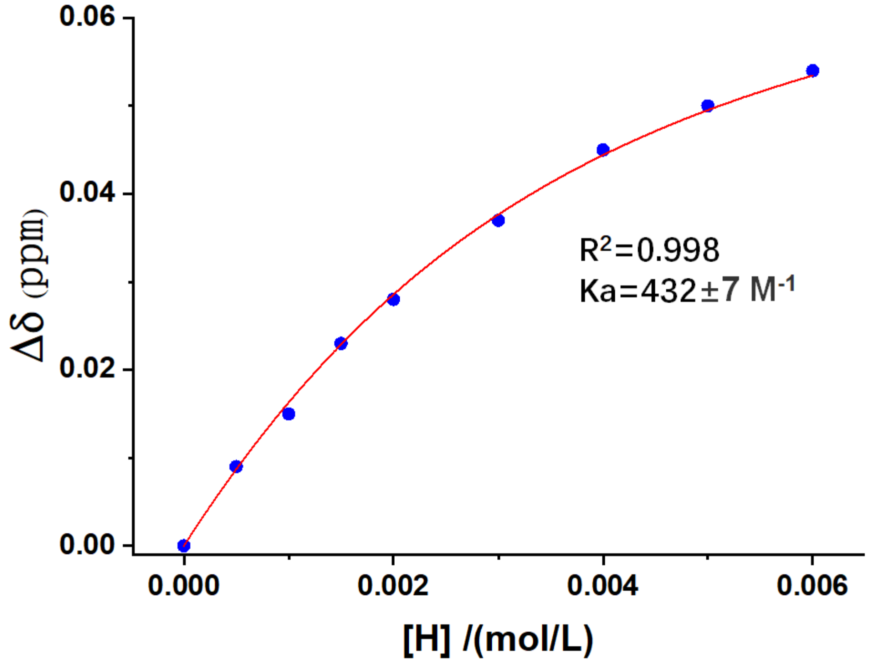

Fig. S23 The chemical shift changes of C_{14} on **6** upon the addition of **1**. The red solid line was obtained from the non-linear curve-fitting using Eq.1. Related to STAR Methods and Figure 3.

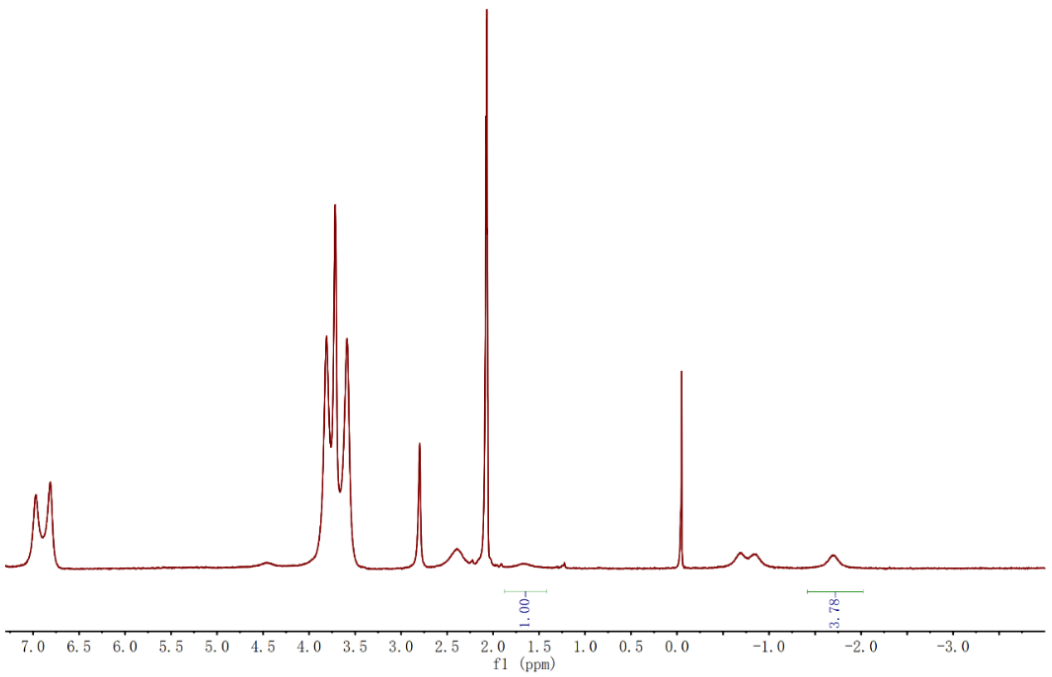

Fig. S24 partial ^{1}HNMR spectrum (400 MHz, $CDCl_3/CD_3COCD_3$ = 3/1, v/v, 298 K) of 2.00 mM **1** and **2**. Related to STAR Methods and Figure 3.

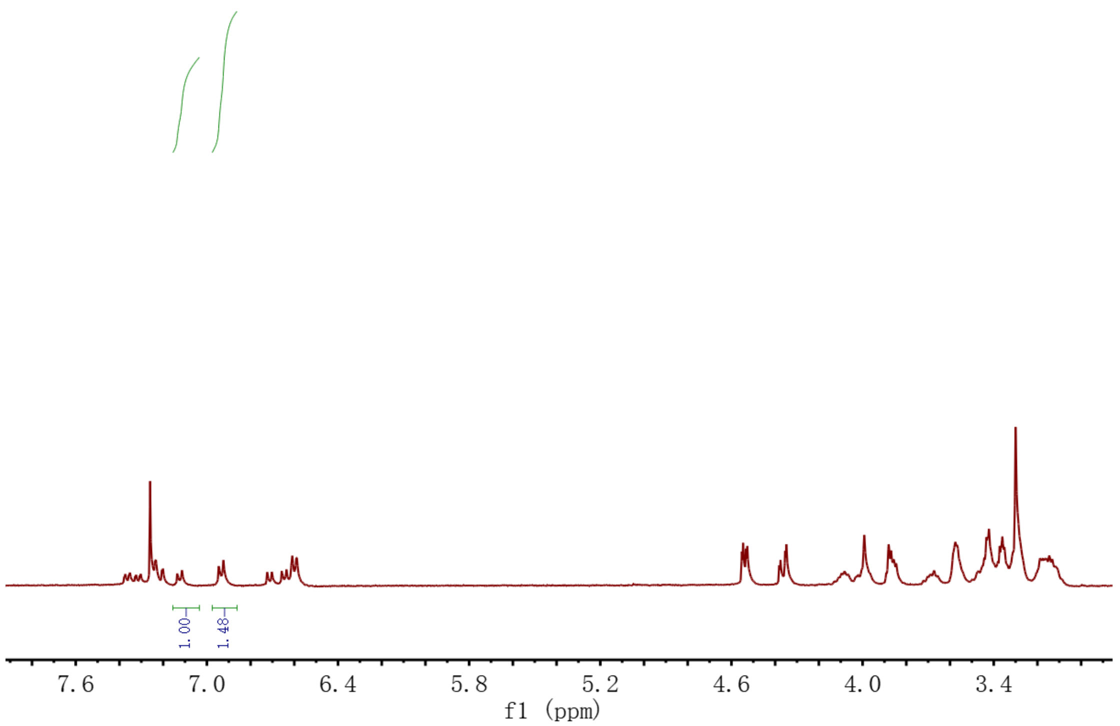

Fig. S25 partial ^{1}HNMR spectrum (400 MHz, $CDCl_3/CD_3COCD_3$ = 3/1, v/v, 298 K) of 5.00 mM **5** and **6**. Related to STAR Methods and Figure 3.

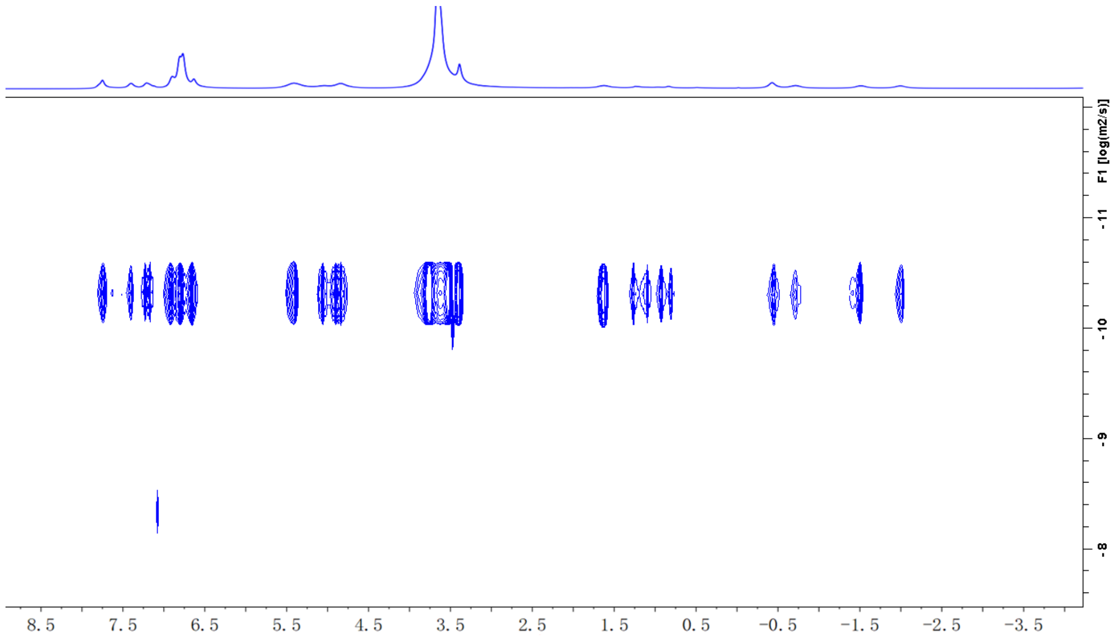

Fig. S26 Representative DOSY spectrum (600 MHz, $CDCl_3$-CD_3COCD_3 = 3/1, v/v, 298 K) of AB_2, the AB_2 concentration is 110 mM. Related to Figure 4.

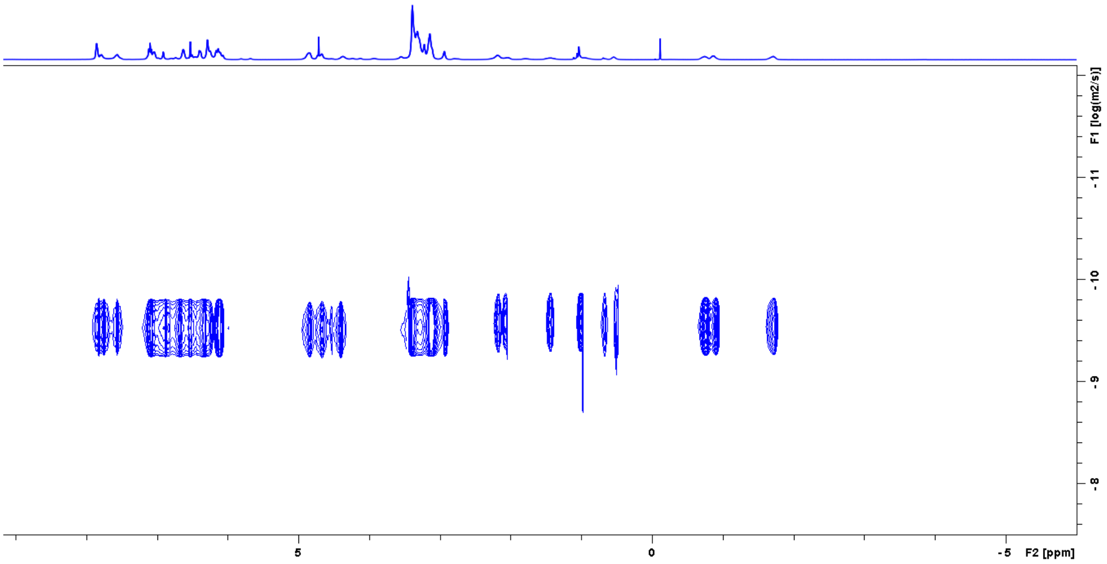

Fig. S27 Representative DOSY spectrum (600 MHz, $CDCl_3$-CD_3COCD_3 = 3/1, v/v, 298 K) of AB_2+CD, the AB_2 concentration is 110 mM. Related to Figure 4.

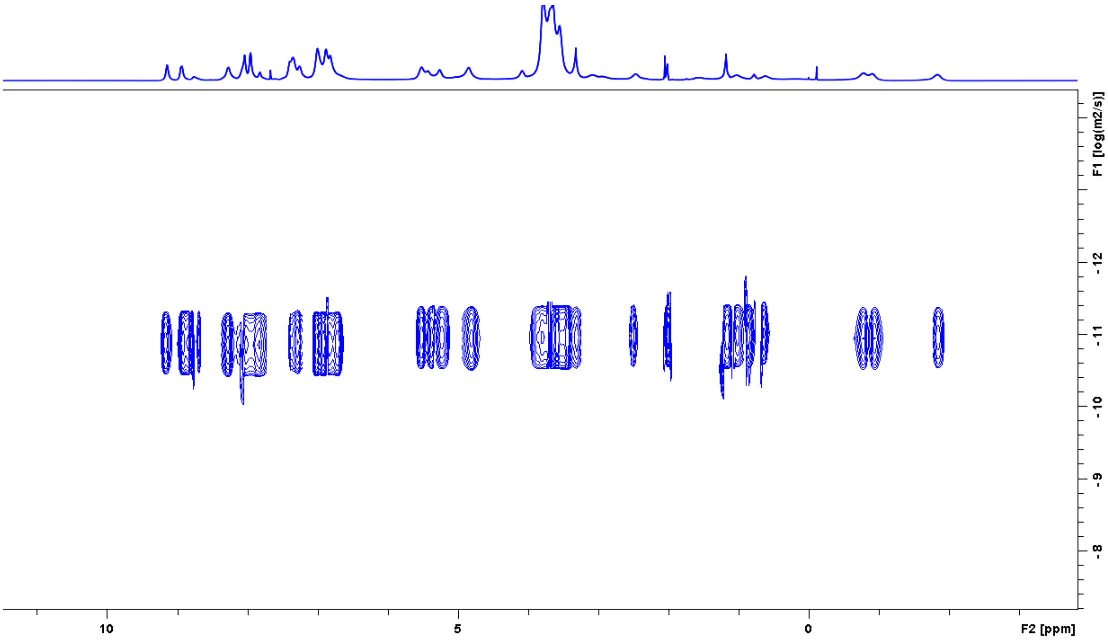

Fig. S28 Representative DOSY spectrum (600 MHz, $CDCl_3$-CD_3COCD_3 = 3/1, v/v, 298 K) of AB_2+CD+ $Zn(OTf)_2$, the AB_2 concentration is 110 mM. Related to Figure 4.

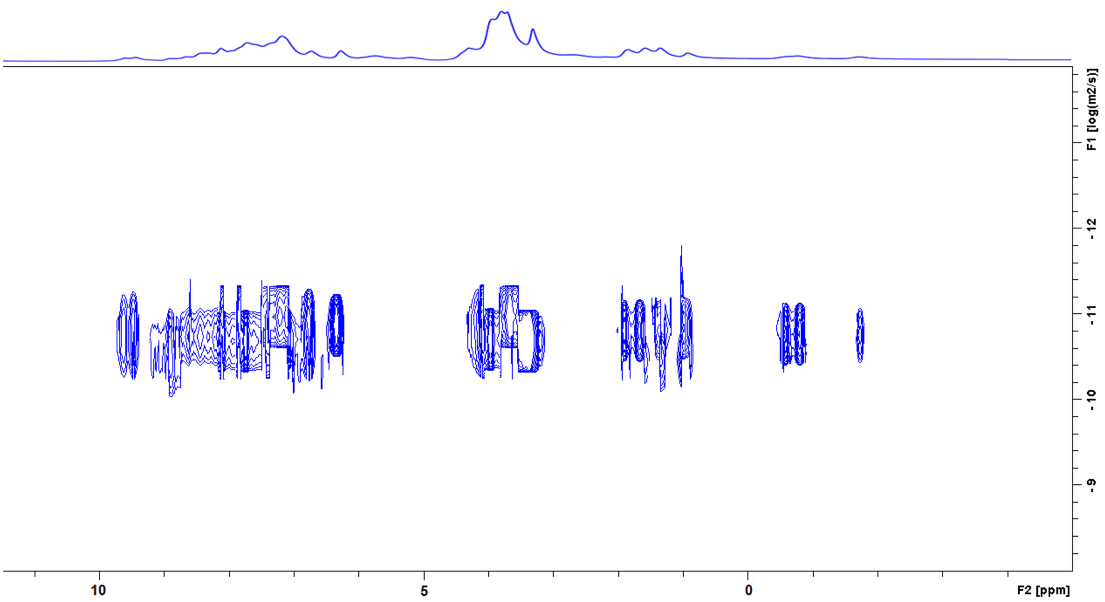

Fig. S29 Representative DOSY spectrum (600 MHz, $CDCl_3$-CD_3COCD_3 = 3/1, v/v, 298 K) of AB_2+CD+EF+$Zn(OTf)_2$, the AB_2 concentration is 110 mM. Related to Figure 4.

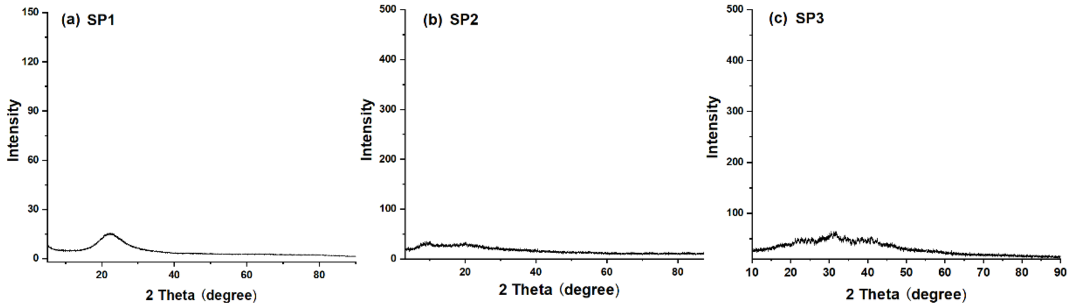

Fig. S30 XRD analysis of samples obtained from SP1, SP2, and SP3. Related to Figure 6.

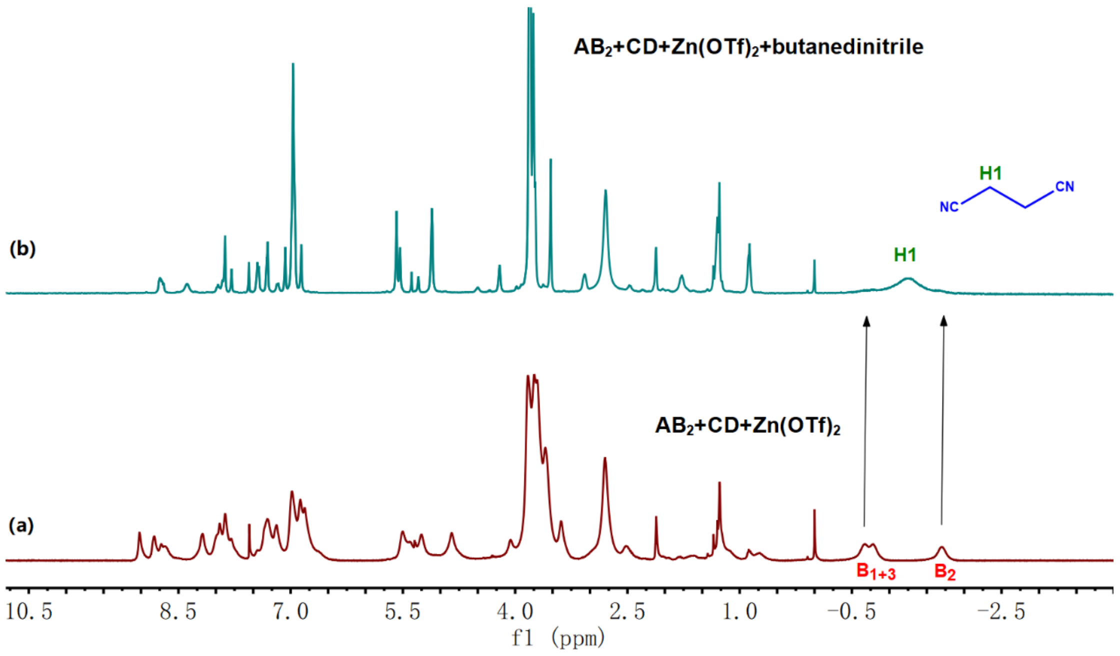

Fig. S31 ^{1}H NMR spectra (400 MHz, $CDCl_3$-CD_3COCD_3 = 3/1, v/v, 298 K) of (a) AB_2+CD+$Zn(OTf)_2$ (40 mM), (b) after the addition of 2 equiv. butanedinitrile. Related to Figure 6.

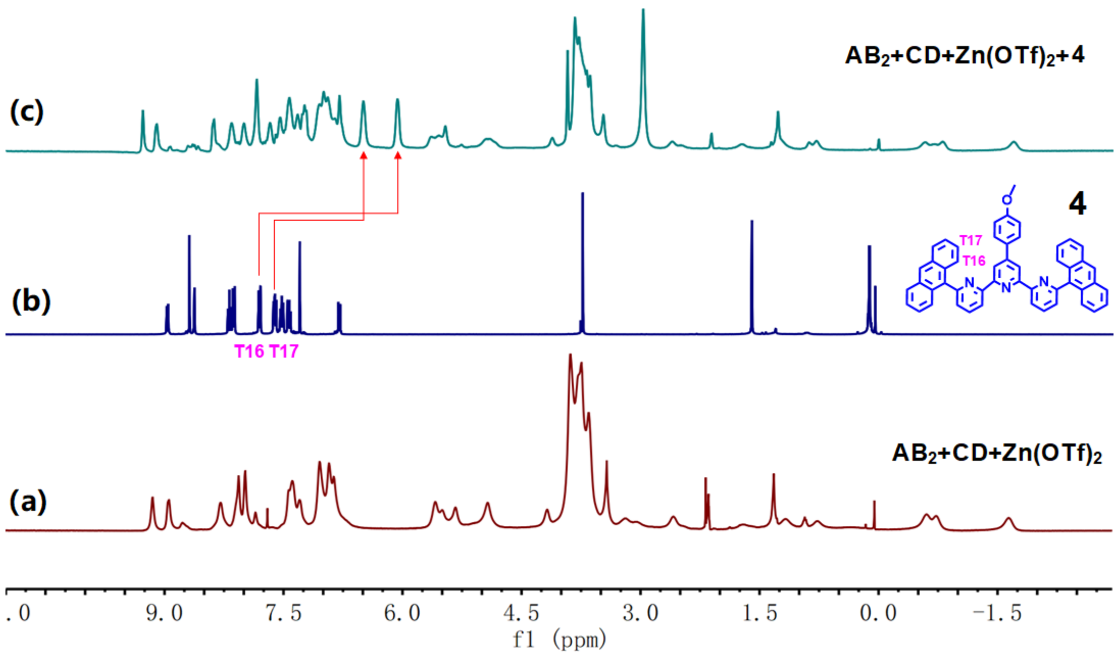

Fig. S32 ^{1}H NMR spectra (400 MHz, $CDCl_3$-CD_3COCD_3 =3/1, v/v, 298 K) of (a) AB_2+CD+$Zn(OTf)_2$ (40 mM), (b) 4, (c) after adding 2 equiv. compound 4 into the solution of AB_2+CD+$Zn(OTf)_2$. Related to Figure 6.

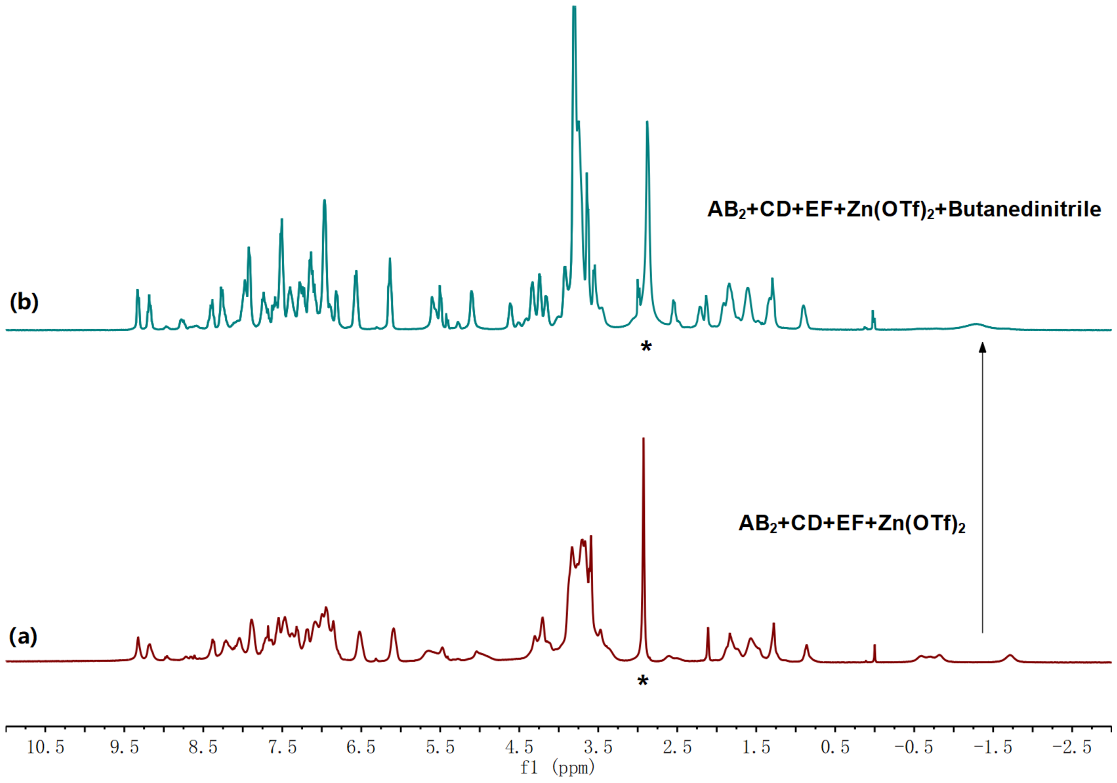

Fig. S33 ^{1}H NMR spectra (400 MHz, $CDCl_3$-CD_3COCD_3= 3/1, v/v, 298 K, 40 mM) of (a) AB_2+CD+EF+Zn$(OTf)_2$, (b) after the addition of 2 equiv. butanedinitrile. Related to Figure 6.

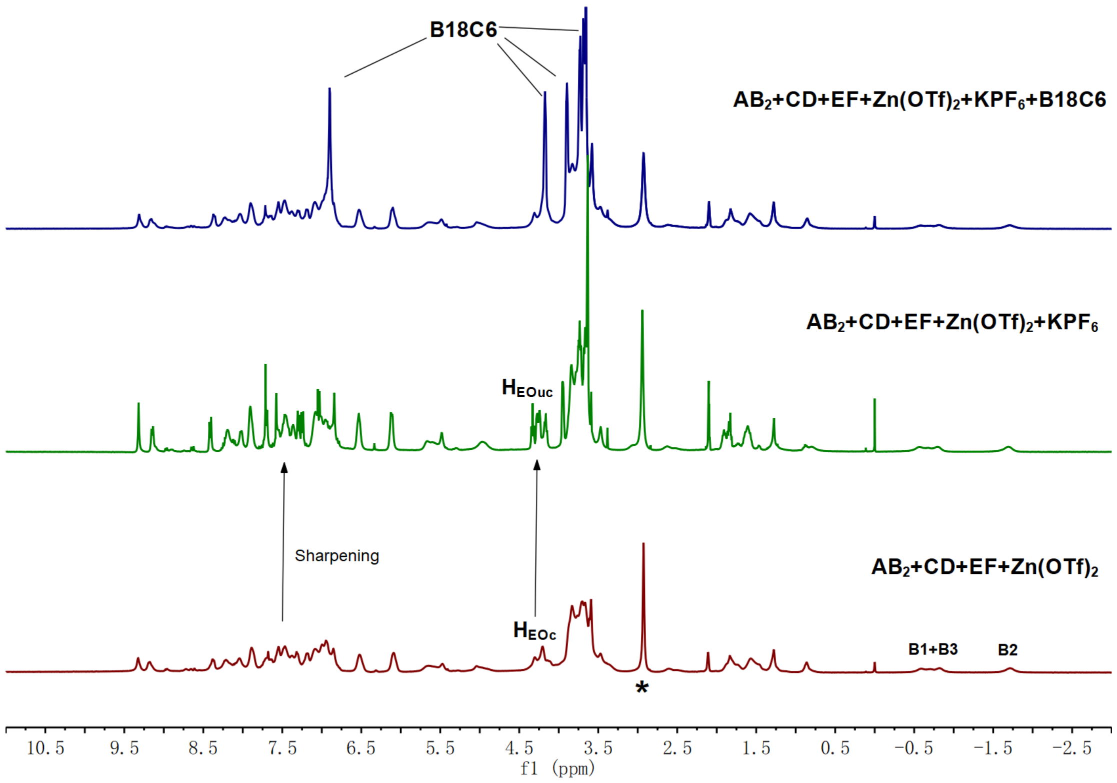

Fig. S34 ^{1}H NMR spectra (400 MHz, $CDCl_3$-CD_3COCD_3= 3/1, v/v, 298 K, 40 mM) of (a) AB_2+CD+EF+Zn$(OTf)_2$, (b) after the addition of 1 equiv. KPF_6, and (c) after the addition of 1.1 equiv. B18C6. Peaks of complexed monomers and uncomplexed monomers were designated as c and uc, respectively. Related to Figure 6.

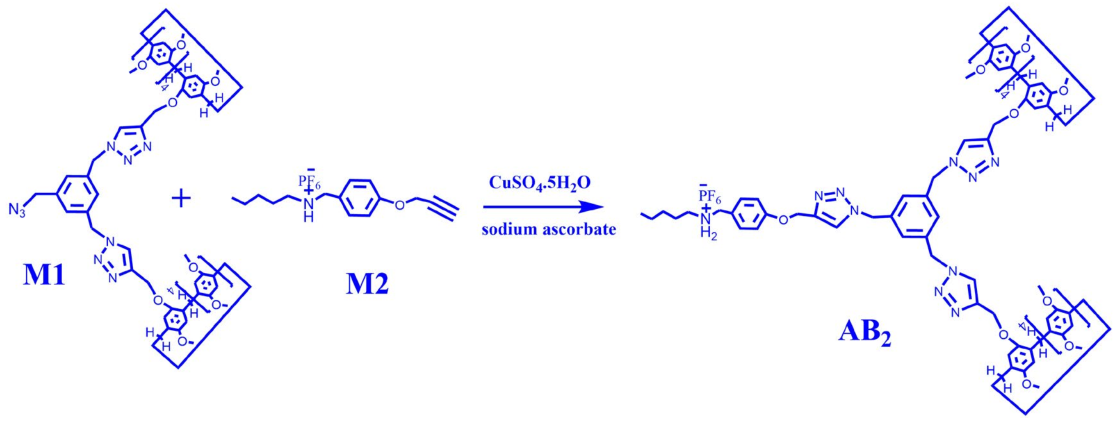

Scheme S1 The synthesis of monomer AB_2. Related to STAR Methods.

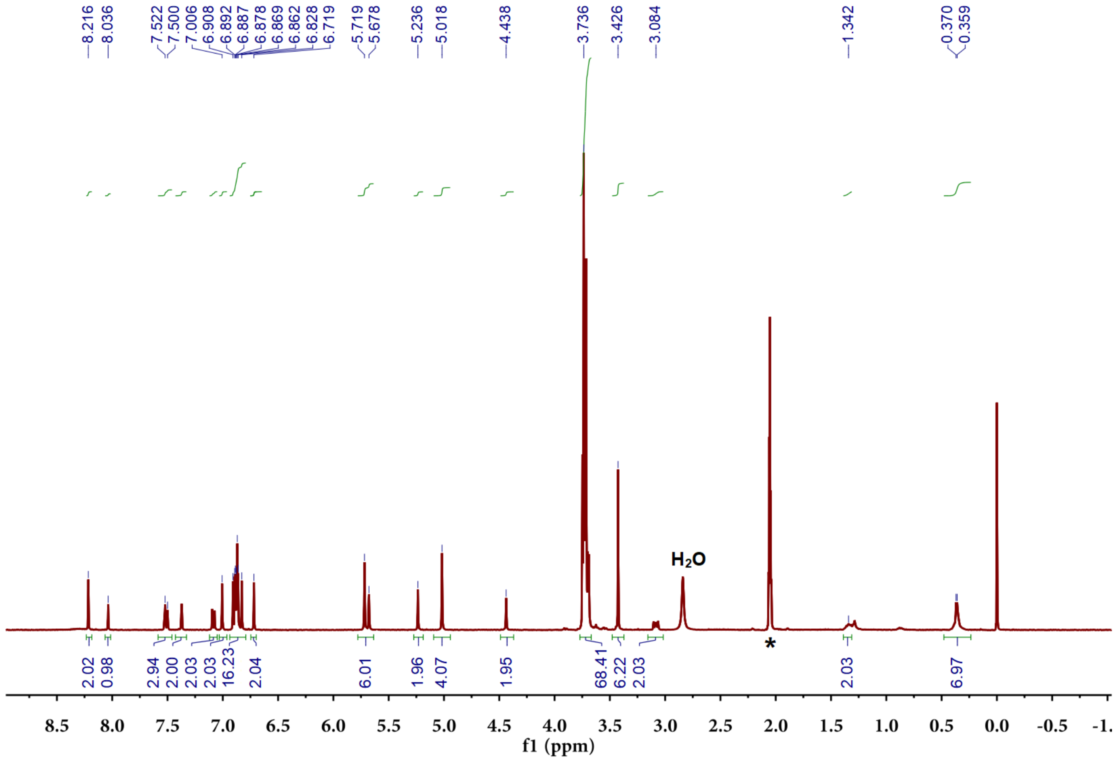

Fig. S35 ^{1}H NMR spectrum (400 MHz, $CDCl_3$,298K) of compound AB_2. Related to STAR Methods.

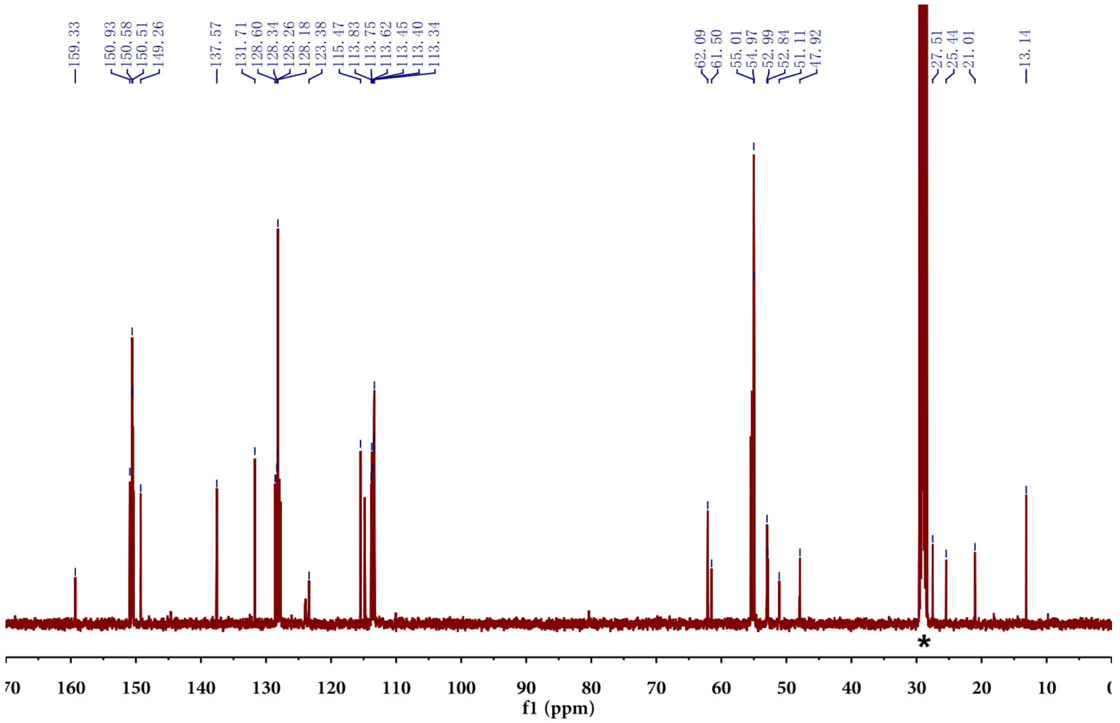

Fig. S36 ^{13}C NMR spectrum (100 MHz, $CDCl_3$, 298 K) of compound AB_2. Related to STAR Methods.

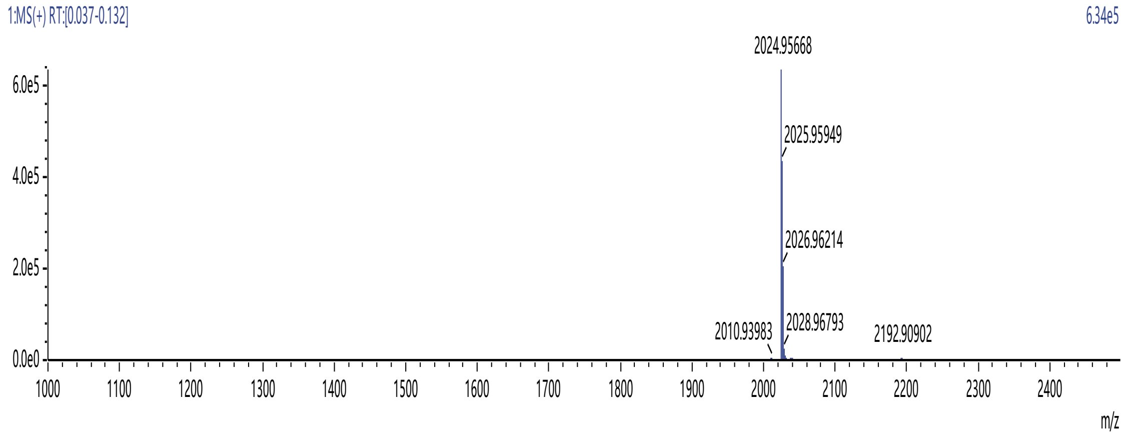

Figure S37 High-resolution MALDI-TOF-MS spectrum of compound AB_2. Related to STAR Methods.

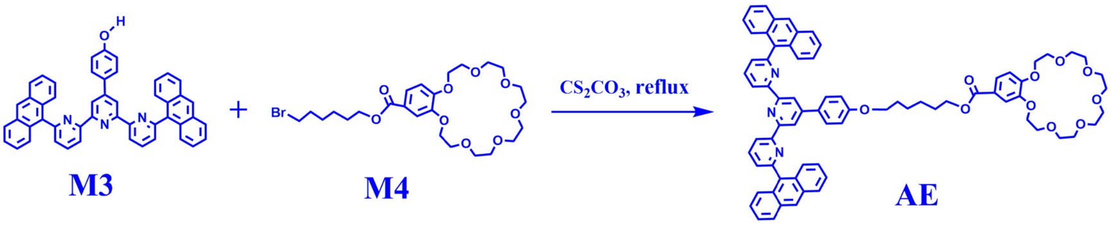

Scheme S2 The synthesis of monomer AE. Related to STAR Methods.

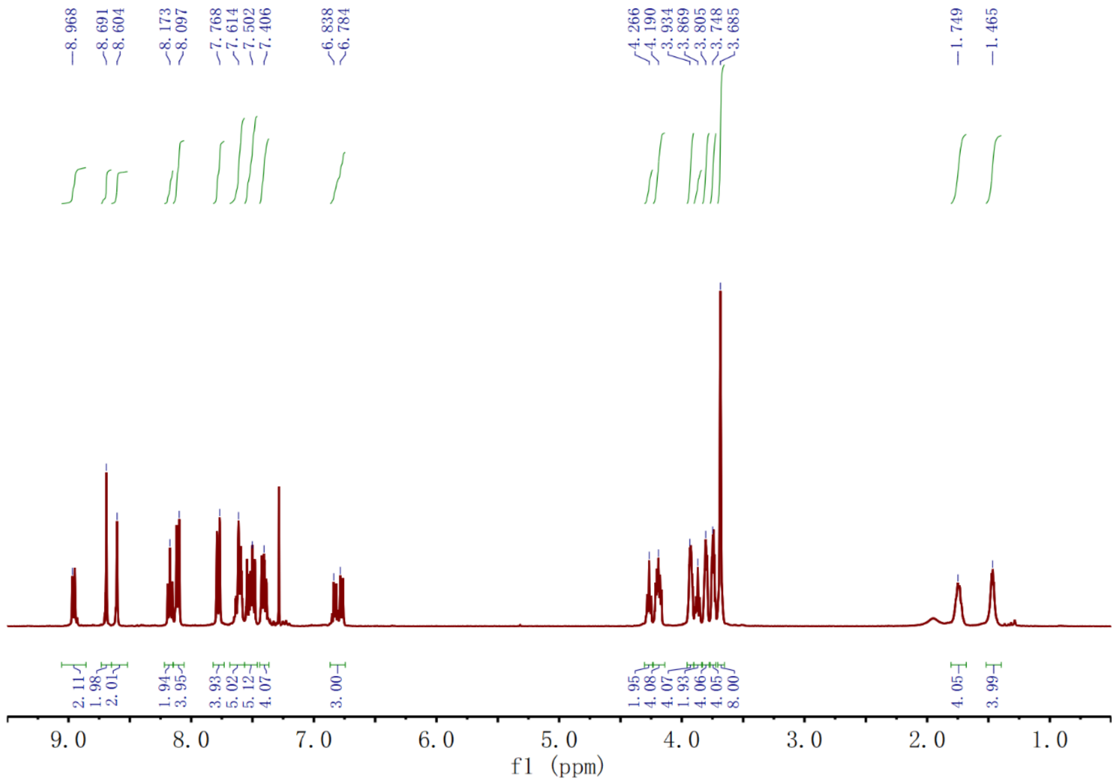

Fig. S38 ^{1}H NMR spectrum (400 MHz, $CDCl_3$, room temperature) of **AE**. Related to STAR Methods.

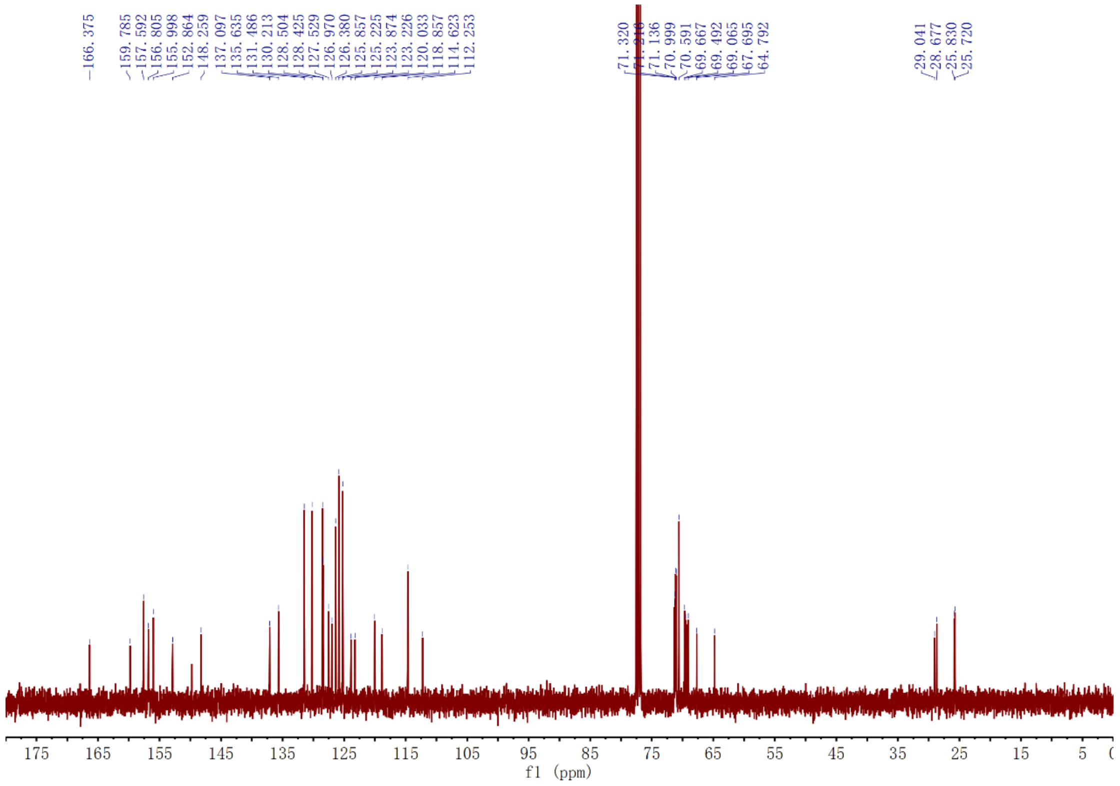

Fig. S39 ^{13}C NMR spectrum (100 MHz, $CDCl_3$, room temperature) of **AE**. Related to STAR Methods.

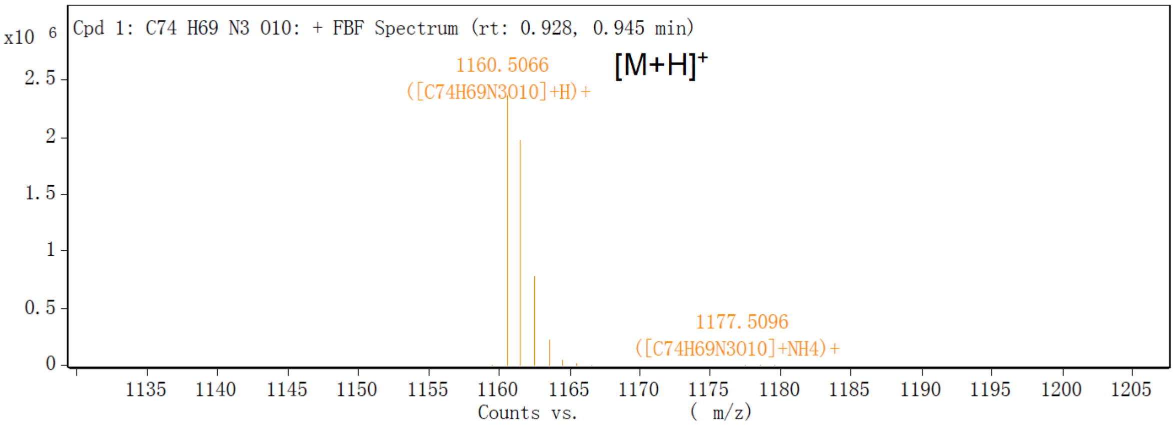

Fig. S40 High-resolution MALDI-TOF-MS of compound **AE**. Related to STAR Methods.
